# Animation of natural scene by virtual eye-movements evokes high precision and low noise in V1 neurons

**DOI:** 10.3389/fncir.2013.00206

**Published:** 2013-12-27

**Authors:** Pierre Baudot, Manuel Levy, Olivier Marre, Cyril Monier, Marc Pananceau, Yves Frégnac

**Affiliations:** Unité de Neuroscience, Information et Complexité, UPR 3293 Centre National de la Recherche ScientifiqueGif-sur-Yvette, France

**Keywords:** natural visual statistics, visual cortex, sensory coding, intracellular membrane potential dynamics, eye movements, reliability

## Abstract

Synaptic noise is thought to be a limiting factor for computational efficiency in the brain. In visual cortex (V1), ongoing activity is present *in vivo*, and spiking responses to simple stimuli are highly unreliable across trials. Stimulus statistics used to plot receptive fields, however, are quite different from those experienced during natural visuomotor exploration. We recorded V1 neurons intracellularly in the anaesthetized and paralyzed cat and compared their spiking and synaptic responses to full field natural images animated by simulated eye-movements to those evoked by simpler (grating) or higher dimensionality statistics (dense noise). In most cells, natural scene animation was the only condition where high temporal precision (in the 10–20 ms range) was maintained during sparse and reliable activity. At the subthreshold level, irregular but highly reproducible membrane potential dynamics were observed, even during long (several 100 ms) “spike-less” periods. We showed that both the spatial structure of natural scenes and the temporal dynamics of eye-movements increase the signal-to-noise ratio by a non-linear amplification of the signal combined with a reduction of the subthreshold contextual noise. These data support the view that the sparsening and the time precision of the neural code in V1 may depend primarily on three factors: (1) *broadband input spectrum*: the bandwidth must be rich enough for recruiting optimally the diversity of spatial and time constants during recurrent processing; (2) *tight temporal interplay of excitation and inhibition*: conductance measurements demonstrate that natural scene statistics narrow selectively the duration of the spiking opportunity window during which the balance between excitation and inhibition changes transiently and reversibly; (3) *signal energy in the lower frequency band*: a minimal level of power is needed below 10 Hz to reach consistently the spiking threshold, a situation rarely reached with visual dense noise.

## Introduction

The potential capacity of the brain in coding external events depends on both the *irregularity* of the evoked neuronal responses (variability over time) and their *reliability* (inverse of stimulus-locked variability across trials). To carry information about external dynamical events (stimuli), neural activity must indeed fulfill two conditions. *First*, it must vary over time. However, abrupt changes in firing do not necessarily signal the presence of an input, since spike pattern irregularity in time is characteristic of the ongoing dynamics of cortical networks. In such networks, feedback and re-entrant connections largely outnumber feed-forward inputs and favor the persistence of reverberating activity. Notwithstanding the intrinsic stochastic nature of the firing process itself, recurrent connectivity is sufficient to generate a level of irregularity similar to that observed *in vivo*, both in the ongoing and evoked modes, as shown by deterministic generic cortical-like network models (van Vreeswijk and Sompolinsky, 1996; Vogels et al., 2005; Marre et al., 2009). *Second*, information transfer should be reliable on a trial-by-trial basis. In order to extract the reliability of signal transmission for repeated stimulus presentations, measures of the signal and noise (contextual, since specifically dependent on each full field stimulation condition), have to be achieved relative to trial onset, both at the synaptic and spiking levels.

A standing issue, concerning sensory processing efficiency in the early visual system, is asserting the functional impact of these two types of neural variability, respectively as a function of time and on a trial-by-trial basis. In single neocortical neurons *in vitro*, because of the reduced recurrence in the network, time variability of synaptic bombardment is minimal. In such test condition, neuronal integration at the soma elicits reliable and precise spike responses to the repeated intracellular injection of temporally irregular current waveforms, whereas responses to identical current steps show a high level of variability (Mainen and Sejnowski, 1995; Nowak et al., 1997). However, this result does not seem to be immediately transposable to the *in vivo* case, where both time variability of the synaptic bombardment and trial-by-trial variability of evoked responses are much higher. Indeed, most *in vivo* extracellular recordings lead to the observation of supra-Poisson spike count variability in response to bars and gratings (Heggelund and Albus, 1978; Dean, 1981). Sub-Poisson cortical responses have also been reported in higher cortical areas (Maimon and Assad, 2009), but more rarely in early sensory cortex. Reduced variability was observed in V1 only in specific conditions in the thalamo-cortical recipient layer 4 cells, during dense levels of firing evoked by optimal drifting gratings, when the spiking rate becomes regularized by the interspike refractory period (Kara et al., 2000; but see Gur and Snodderly, 2006). Although more recent work in the behaving animal points to the importance of decision and other cognitive processes in shaping correlated variability (Nienborg et al., 2012), the most commonly accepted interpretation of variable responses in the anesthetized animal or *in vitro* slices is that ongoing activity acts as an independent source of “noise”, which adds to the deterministic sensory signal and corrupts its propagation. This linear “Signal + Noise” model has been extensively used to justify a coding scheme based on averaging across time (rate coding) and/or neuronal assemblies (population code) (review in Shadlen and Newsome, 1998). It has been applied at different scales of integration ranging from single neurons (Azouz and Gray, 1999; Deweese and Zador, 2004) to cortical columns (Arieli et al., 1996). This view, which assumes independency between Signal and Noise, has been however recently disputed: an extensive review of numerous independent electrophysiological cortical studies shows convincingly that variability of evoked responses is not stationary during the trial time-course and goes through a minimal value at some fixed delay following the presentation onset of most sensory stimuli (Churchland et al., 2010; see Monier et al., 2003 for a mechanism).

A second issue is the dependence of the variability of neuronal responses on the nature itself of the stimulus: it may be that stimuli having richer and/or more natural statistics are required to constrain the network dynamics and that the presence of higher input frequencies is needed to explore the upper limit of coding efficiency (Borst and Theunissen, 1999). Indeed, at subcortical stages, fast-varying noise and natural scenes elicit irregular, precise and reliable spike responses (retina: Berry and Meister, 1998; LGN: Reinagel and Reid, 2000; Lesica and Stanley, 2004; Lesica et al., 2006; Butts et al., 2007; see also De Ruyter van Steveninck et al., 1997). The highest stimulus-locked spiking precision in the LGN is expressed when stimulating with dense noise statistics (Butts et al., 2007). At the cortical level, fast random motion variations evoke precise but still Poisson discharges in MT (Buracas et al., 1998). However, the effect of enriched spatio-temporal statistics, such as experienced during normal sensory-motor exploration, remains only partially documented in V1 (Baddeley et al., 1997; Vinje and Gallant, 2000, 2002). The stimulation of the surround of V1 cells with natural scenes has been shown to enhance sparseness (Vinje and Gallant, 2000, in awake behaving monkeys) as well as reliability (Haider et al., 2010; Herikstad et al., 2011, in anesthetized cat). More generally, the global sensory context (full field, dense dynamic stimulation) in which one probes visual receptive fields appears determinant in shaping their spatio-temporal profiles (Fournier et al., 2011). These different findings advocate for a quantitative comparison, in the same cortical cell, of the reliability of the responses and the time precision of the neural code as a function of the statistics of the full field sensory flow.

**Figure 1:**
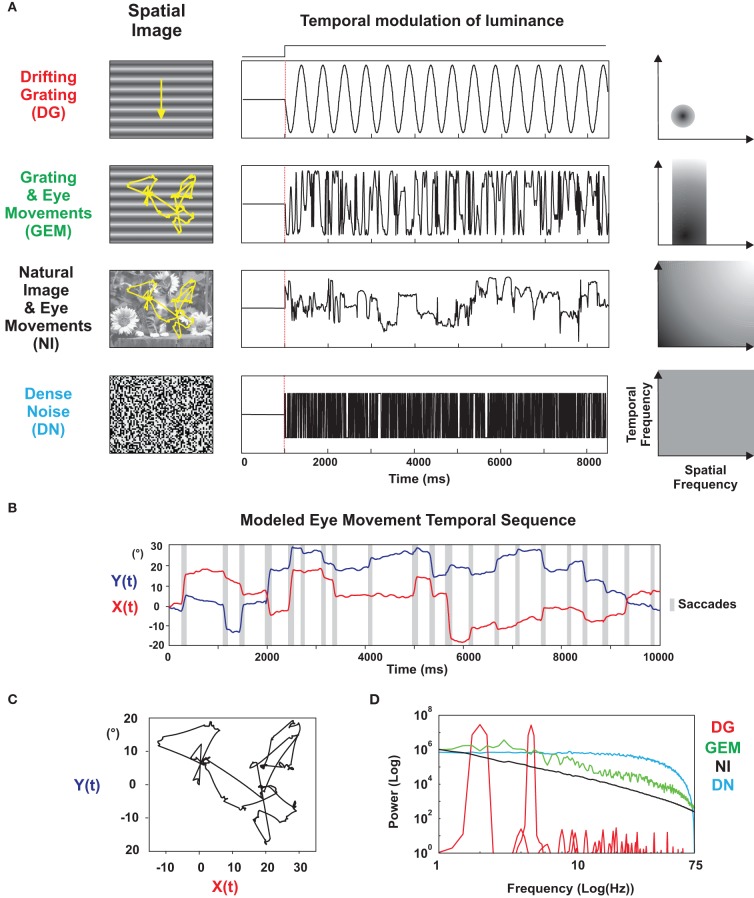
**Parametrization of stimulus statistics. (A)** *Left column*: stimulus set presented to each intracellularly recorded cell. From top to bottom (by increasing order of complexity): (1) Drifting Grating (DG): a sinusoidal grating with optimal spatial frequency and orientation, drifting at optimal temporal frequency; (2) Grating and Eye-movements (GEM): the same grating animated by a trajectory simulating the dynamics of eye-movements; (3) Natural Image (NI) animated with virtual eye-movements; (4) Dense Noise (DN) of high spatial and temporal definition. The variances of the luminance profiles were equalized between stimuli. The presentation was full-field and monocular (through the dominant eye). *Middle column*: temporal variation of the luminance in a given pixel for each stimulus. *Right column*: schematic spatio-temporal spectrum (*ft*, *fx*) corresponding to each stimulus. **(B)** Temporal profile of the *X* and *Y* coordinates of the modeled eye-movement sequence. Saccadic episodes are indicated by a shaded box. **(C)** Scanpath generated by the modeled eye-movement sequence. The natural scene image is centered on the RF center at the start of the animation and the same displacement pattern is applied to all cells (“frozen” protocol). **(D)** Average of the Power spectrum of the luminance variation observed in one pixel for each stimulus condition.

In order to quantify the dependence of the trial-to-trial variability of cortical processing on sensory statistics, we recorded intracellularly visual responses in area 17 of anaesthetized and paralized cats and we compared the visual responses of the same neuron to a set of 4 different full field stimuli of calibrated spatial and temporal properties in the primary visual cortex of the anaesthetized and paralyzed cat. These stimuli included both classical artificial stimuli used to probe neuronal selectivity [drifting grating (DG), dense noise (DN)], and more complex stimuli [grating (GEM) and natural image (NI), both animated with the same natural temporal statistics] that aimed at mimicking as best as possible the global retinal flow received during the exploration of the natural environment (Figure 1A). The visuomotor interaction was simulated by imposing, in the paralyzed condition, shifts and drifts of a static frame (grating or natural scene) which reproduced the kinematics of a realistic ocular scanpath (Figures 1B–D). A time-frequency wavelet analysis of subthreshold membrane potential (*Vm*) waveforms was used to measure the reproducibility of the responses and infer the instantaneous synchrony state of presynaptic afferents across stimuli (see Materials and Methods and Figure 5). We report here that the retinal flow statistics imposed by simulated eye-movements evoke reliable, non-linear responses in V1, and that sparse spike responses to natural stimuli arise from irregular but highly reproducible *Vm* trajectories. Additional conductance measurements show that the neural code reliability revealed by natural scene statistics relies on sparse and phasic changes in the balance between excitation and inhibition, and a reduction of stimulus-locked noise in subthreshold *Vm* dynamics. Both effects contribute to the optimal shaping of the temporal width of the spike opportunity window, and to the temporal precision of the code for animated natural scenes.

## Materials and methods

### Preparation and *in vivo* recordings

All surgical procedures and animal experimentation were performed in conformity with national (JO 87-848) and European (86/609/CEE) legislations on animal experimentation, and strictly following the recommendations of the Physiological Society, the European Commission and NIH. The level of anaesthesia was monitored by regularly checking the pupillary state (during surgery before atropine instillation) and the stability of the physiological parameters (ECG, EEG, expired CO2) in response to a standard paw-pinching test. In addition, ECG, EEG, expired CO2 and body temperature were continuously monitored and stabilized during all the experiment. Cells in the primary visual cortex of anaesthetized (Althesin) and paralyzed adult cats were recorded *in vivo* using sharp electrode recordings (average Vrest = −67 mV, 0 nA) as described elsewhere (Monier et al., 2003). The electro-corticogram (ECoG) was simultaneously recorded using silver electrodes positioned homotopically or close to the recording site. Data processing and visual stimulation protocols used in-house software (G. Sadoc, Elphy, Biologic CNRS-UNIC/ANVAR).

### Visual stimulation

Stimuli were displayed on a 21” CRT monitor with a 1024 × 768 pixel resolution and a 150 Hz refreshing rate, with a background luminance of 12 cd/m^2^. Receptive Fields (RFs) were mapped using sparse noise with screen refreshing every 7 frames (47.7 ms) and classical tunings (orientation, phase, spatial frequency) were determined by automated exploration. The viewing distance was set to 57 cm, and the movie covered 20° of viewing solid angle. The mean luminance and contrast of each movie were equalized so that they differed only in their higher-order statistics. Each full field movie was presented at least 10 times for a 10 s duration. For the natural-like condition, we used a high definition NI (2048 × 1536 pixels) animated with a virtual eye-movement sequence (see below). White noise consisted of a dynamic sequence (13.3 ms refresh period) of high spatial definition (50 * 50 pixels of 0.39°) binary dense noise.

### Simulation of virtual eye-movements

Eye-movements are classically decomposed into intermittent ballistic movements, i.e. saccades, of large but variable amplitude, separated by fixation episodes. During fixation, the mean position of the eye drifts slowly in time, with superimposed very low amplitude tremors at high frequency (40–100 Hz range) as well as microsaccades. In order to simulate in a realistic way the continuous changes imposed by eye-movements during natural scanning of visual scenes, we built a model of the retinal flow (example in Figure 1C) whose kinematic parameters were fitted on the basis of measurements previously made in the freely behaving cat (Pritchard and Heron, 1960; Collewijn, 1977; Olivier et al., 1993). A more detailed description follows:

#### Saccades

The saccade amplitudes and intersaccadic intervals were chosen randomly from the distribution established for saccadic and head gaze movements in the freely behaving cat (Collewijn, 1977). An estimate of the duration of the saccade (*D_S_*) was made by using the best linear fit between saccadic amplitude (*A_s_*) and duration:

(1) $$D_{S}=1.9\times A_{S}+63$$

where *D_S_* is expressed in ms and *A_s_* in steradian degrees (°) of visual angle. The saccadic spatio-temporal profile was modeled by the following sigmoidal function *F*(*t*):

(2) $$F(t)=-\lambda A_{S}+\left(A_{S}+2\lambda A_{S}\right)/(1+e^{(-2-\lambda )/\left(D_{S}\left(D_{S}/2-t\right)\right)})$$

where λ is a constant threshold fixed at 5%. The direction of the movement was chosen randomly from a uniform [0°, 360°] distribution. Since most saccadic paths present small drifts of directional angle during their execution (Yarbus, 1967; Rucci and Desbordes, 2003), an *ad-hoc* sinusoidal variation of direction during the drift path was fitted to real recordings:

(3) $$f(t)=\Delta \theta \sin\left(2.\pi t.\tau /D_{S}\right)$$

where the amplitude of direction change (Δθ) was chosen randomly from a uniform distribution between 0° and 4°, and the fraction of during which it operated (τ) was chosen randomly between 0.5 and 1 (relative to the full saccade duration).

#### Drifts

The drift amplitude (*A_D_*) was chosen randomly from a Gaussian distribution with a mean of 1.21° and a standard deviation of 0.63°. The duration (*D_D_*) was derived from the best linear fit with *A_D_*. These parameter values were taken from measures in the behaving cat (Olivier et al., 1993):

(4) $$D_{D}=41.7\times A_{D}+53.7$$

where *D_D_* is expressed in ms and *A_D_* in °.

The direction of drift movement was chosen randomly from a uniform [0°, 360°] distribution. The same *ad-hoc* sinusoidal variation of direction during the drift path (Equation 3) was fitted to real recordings, but with direction change chosen randomly between 0 and 29°.

#### Tremors (during drifts)

Tremor eye-movements are typically of miniature amplitude, ranging from 0.001 to 0.017° [0.006–0.013° in Rucci and Desbordes (2003); 0.005° in Ratliff and Riggs (1950); 0.001°–0.004° in Ditchburn and Ginsborg (1952); Ditchburn (1973)], with a mean amplitude of 0.007° in the cat (Pritchard and Heron, 1960). The simulation of tremor was constrained by the spatial discretization of the screen (1024 × 768 pixels) and the imposed viewing distance (57 cm). In the present experiments, the smallest programmable distance between two neighboring pixels was 0.039°. For spectral characteristics, we chose to remove most of the tremor energy due to low amplitude micro-movements while keeping its highest amplitude components. This was achieved by using a white noise signal through a Bessel filter, between 40 and 80 Hz (Eizenman et al., 1985). The sequence movement thus obtained was then discretized, using only three possible inter-pixel amplitude values (−1, 0, 1), and low-pass filtered.

***Microsaccades*.** Microsaccades are particularly rare in cats (Körding et al., 2001) and our modeled “frozen” eye movement sample sequence contains only three of them positioned at the end of a tremor. Their amplitude was chosen randomly from a Gaussian distribution with mean and standard deviation both set to 1°, thresholded for amplitudes less than 0.02°, as found in humans (Ditchburn, 1973). An estimate of their duration (*D_ms_*) on the basis of Ditchburn's observations in humans (Ditchburn, 1973), was given by the best linear fit between micro-saccadic amplitude *A_ms_* and duration:

(5) $$D_{ms}=2.25\times A_{ms}+20$$

where *D_ms_* is expressed in ms and *A_ms_* in ° of visual angle.

The microsaccadic spatio-temporal profile, direction and variation of angle during the microsaccade were modeled as for saccades.

### Reliability, precision, and sparseness of subthreshold and spiking responses

The reliability and the precision of the responses were measured by fitting a Gaussian function to the cross-correlation (CC)—across trials—of the spiking responses, and, extending a previous analysis of spiking responses (Butts et al., 2007), of subthreshold membrane potential responses (after spike filtering). The reliability was given by the CC peak amplitude at time zero, and the temporal precision by the standard deviation of the Gaussian fit. Classical measures of irregularity in the spiking discharge were performed using the Coefficient of Variation of the Interspike Interval distribution (ISI CV). To quantify sparseness we used a non-parametric index (Vinje and Gallant, 2000):

(6) $$S=\left(1-{\left(\sum r_{i}/n\right)}^{2}/\sum \left(r_{i}^{2}/n\right)\right)/\left(1-\left(1/n\right)\right)$$

where *r_i_* is the response to the *i*th frame of a movie (averaged across trials) and *n* is the number of movie frames. *S* values (expressed in % in Figure 4) range between 0 (0%) for a dense code, and 1 (100%) for a sparse code. The duration of the frame movie is 13.3 ms. The sparseness index was calculated also as a function of bin width values ranging between 1 and 100 ms (with a step of 1 ms).

For the calculation of the Fano Factor, spike counts were computed by dividing the time axis in successive 13.3 ms bins. We then computed the variance (across trials) and the mean of the spike count. A scatter plot of the variance vs. the mean was compiled, with one point per time window, for all the duration of the stimulation (10 s). The raw Fano factor was given by the slope of the regression line relating the variance to the mean.

### Stimulus-locked time-frequency analysis

Spike trains and subthreshold *Vm* waveforms were convolved for each trial (one repeat of the same movie clip) with an array of complex-valued normalized Gabor functions Ψ_*f*_ (τ )

(7) $$\Psi_{f}(\tau )=(a/\sqrt{f})\cdot \exp(-2.\pi .i.f.\tau )\cdot \exp\left(-\frac{\tau^{2}}{\sigma^{2}}\right)$$

where *a* is a constant such that the energy of the wavelet is equal to 1. To improve the readability of the time-frequency representation, the Gabor decomposition presented here is largely oversampled: the Gabor filter bank is non-orthogonal, with wavelet frequencies ranging from 1 to 75 Hz (with incremental step of 1 Hz), and a temporal sampling period of 1 ms. To achieve a fine temporal resolution (important for spike events), the normalized Gabor function had a Gaussian window variance equal to two Gabor periods (σ.*f* = 2). This time-frequency decomposition allows the extraction of Signal power, Noise power, and Signal to Noise ratio (SNR) power (Figure 5). This analysis can be viewed as an extension of the Signal and Noise estimation method proposed by (Croner et al., 1993) to the time-frequency domain.

We define *S*(*t, f*) as the complex result, at time *t* and frequency *f*, of the convolution between the wavelet and the response *X*(*t*) for each trial:

(8) $$S(t,f)=\int X(t-\tau )\cdot \Psi_{f}(\tau )d\tau$$

The Signal power *S*_est_ (*t, f*) of the stimulus-locked waveforms is given by:

(9) $$S_{\text{est}}(t,f)=\left|{\langle S_{i}(t,f)\rangle }_{i}\right|$$

where angular brackets 〈〉 indicate the average across all trials *i* of the wavelet transform in the complex domain and straight brackets indicate the squared modulus.

The Noise power *N*(*t, f*) is measured as the average distance between the individual trial vectors and the average vector of the wavelet transform in the complex domain:

(10) $$N(t,f)={\langle \left|S_{i}(t,f)-{\langle S_{i}(t,f)\rangle }_{i}\right|\rangle }_{i}$$

The Signal to Noise ratio *SNR*(*t, f*) is calculated as:

(11) $$SNR(t,f)=\frac{\left|{\langle S_{i}(t,f)\rangle }_{i}\right|}{{\langle \left|S_{i}(t,f)-{\langle S_{i}(t,f)\rangle }_{i}\right|\rangle }_{i}}=S_{\text{est}}(t,f)/N(t,f)$$

In the case of spike train signals, SNR was assigned a zero value for the times and frequencies when a total absence of activity was observed for all trials (*S_i_* (*t, f*) = 0, ∀ *i*). Signal, Noise, and SNR power spectra are obtained by averaging the squared functions over time:

(12) $$F_{\text{SNR}}(f)=\int_{t_{\text{start}}}^{t_{\text{end}}}{\left(\text{SNR}(t,f)\right)}^{2}/(t_{\text{end}}-t_{\text{start}})dt$$

(13) $$F_{\text{Signal}}(f)=\int_{t_{\text{start}}}^{t_{\text{end}}}{\left(S_{\text{est}}(t,f)\right)}^{2}/(t_{\text{end}}-t_{\text{start}})dt$$

(14) $$F_{\text{Noise}}(f)=\int_{t_{\text{start}}}^{t_{\text{end}}}{\left(N(t,f)\right)}^{2}/(t_{\text{end}}-t_{\text{start}})dt$$

These measures represent the average energy of the Signal, Noise and SNR at a given frequency.

### Linear prediction

The linear kernel of the subthreshold RF was fitted by least squares regression, on the basis of an exploratory set of stimulus-response correlations obtained during full-field DN mapping. To avoid overfitting, the calculation was limited to a 8*8 pixel square centered on the largest responsive areas of the subthreshold RF. The linear predictions of the *Vm* response (shown in Figures 11, 13) were obtained by convolving the dense noise RF kernel with each of the 4 stimulus movies, re-sampled at the kernel resolution. RF estimation and validation were realized on different data sets. To compare the energy in the predicted (*Vm*_pred_) and observed (*Vm*_obs_) waveforms, we calculated for each stimulus condition a Static Gain Factor (SGF) as:

(15) $$\text{SGF}=\sigma^{2}\left(Vm_{\text{pred}}\right)/\sigma^{2}\left(Vm_{\text{obs}}\right)$$

When the correlation is high, this gain factor can be interpreted as quantifying the degree of static (space- and time-invariant) suppression that was observed independently of the quality of the linear fit.

### Predicted, expected, and shuffled coherences

The coherence Coh(*f*) measures the degree of linear relationship between two signals s_1_(*t*) and s_2_(*t*) in the Fourier space, and is defined by:

(16) $${\text{Coh}}_{S_{1},S_{2}}(f)=\frac{|<S_{1}(f).S_{2}^{*}(f){>}_{t}{|}^{2}}{<|S_{1}(f){|}^{2}{>}_{t}.\text{​}<|S_{2}(f){|}^{2}{>}_{t}}$$

where *S*_1_ and *S*_2_ are the Fourier transforms of *s*_1_ and *s*_2_. The angular brackets symbolize window averaging (1 s-long Hann windows shifted by 1 s steps in the present study). The coherence equals one for linearly related signals, and decreases below one when the signals are non-linearly related, and/or corrupted by noise. The reliability and the linearity of the *Vm* subthreshold dynamics were characterized by the expected [Coh_Exp_(*f*)] and the predicted [Coh_Pred_(*f*)] coherences, respectively (van Hateren and Snippe, 2001). The coherence between the average response and its linear prediction, Coh_Pred_(*f*), was compared to the maximal coherence that can be reached given the observed neuronal noise level, Coh_Exp_(*f*). To obtain the Coh_Exp_(*f*) we averaged the *n* (*n* = number of stimulus repetitions) coherences computed individually between each trial response and the mean of all other trials.

Intracellular recordings *in vivo* are often of limited duration and the number of repetition of each stimulus may produce biases in the estimations of Coh_Exp_(*f*) and Coh_Pred_(*f*). To control for these biases, we also computed the shuffled coherence Coh_Shuf_(*f*) the same way as Coh_Exp_(*f*). The only difference was that individual trials were time-shifted relative to each other beforehand (trial one by 1 s, trial two by 2 s, …), so that coherences were computed between signals recorded at non-overlapping stimulus presentations. Coh_Shuf_(*f*) provided a baseline to the estimated Coh_Exp_(*f*) and Coh_Pred_(*f*).

Finally, the coherence rate *R*_Coh_ quantifies how close the coherence function is to 1 over the entire frequency range:

(17) $$R_{\text{Coh}}=-\sum_{N_{0}}^{N_{f}}\log_{2}(1-\text{Coh}(f))\Delta f$$

Shuffled coherence rates were subtracted from the expected and predicted coherence rates. The ratio of the and Coh_Pred_(*f*) to Coh_Exp_(*f*) measured the proportion of reliable responses explained by linear mechanisms.

### Conductance measurement and decomposition

Data were analyzed using a method based on the continuous measurement of conductance dynamics during stimulus-evoked synaptic response. This method has been described and validated previously with *in vivo* voltage-clamp and current-clamp recordings (Borg-Graham et al., 1998; Monier et al., 2003; see Monier et al., 2008 for a comparison between the two types of recordings). For subthreshold activity study, spike waveforms were removed and replaced by a low-pass filtered template (*fc* < 100 Hz). The smoothing of the *Vm* trajectory was limited in time to the temporal segment defined between the beginning (time at the spike threshold) and the end of the spiking event itself at its threshold. In the present study, the membrane potential was measured in current clamp mode, at three levels of current. Note that, in our recordings, spike activity could be inactivated during the injection of large positive current (+500 pA) and completely suppressed with large negative current (−500 pA). With current-clamp data, the derivative of the *Vm* waveform can no longer be considered as null and conductances were estimated by taking into account the capacitive current passing through the membrane. To estimate conductances, we used the point-conductance model of a single-compartment cell. The excitatory Gexc(*t*) and inhibitory Ginh(*t*) conductances were calculated from a linear system of equations, corresponding each to a distinct level of applied current (3 levels for 4 cells and 2 levels for 2 cells). In order to avoid spiking contamination, only null or negative currents were used, except for one cell (Cell 8 Figure 13) where strong positive current was used to inactivate spike initiation:

(18) $$\begin{array}{l} {\text{G}}_{\text{inh}}(t)(V_{m}^{1}(t)-E_{\text{inh}})+G_{\text{exc}}(t)(V_{m}^{1}(t)-E_{\text{exc}})\\ =I_{\text{inj}}^{1}-C_{m}\frac{dV_{m}^{1}(t)}{dt}-G_{leak}(V_{m}^{1}(t)-E_{\text{leak}})\\ {\text{G}}_{\text{inh}}(t)(V_{m}^{2}(t)-E_{\text{inh}})+G_{\text{exc}}(t)(V_{m}^{2}(t)-E_{\text{exc}})\\ =I_{\text{inj}}^{2}-C_{m}\frac{dV_{m}^{2}(t)}{dt}-G_{\text{leak}}(V_{m}^{2}(t)-E_{\text{leak}})\\ {\text{G}}_{\text{inh}}(t)(V_{m}^{3}(t)-E_{\text{inh}})+G_{\text{exc}}(t)(V_{m}^{3}(t)-E_{\text{exc}})\\ =I_{\text{inj}}^{3}-C_{m}\frac{dV_{m}^{3}(t)}{dt}-G_{\text{leak}}(V_{m}^{3}(t)-E_{\text{leak}}) \end{array}$$

where *C_m_* denotes the membrane capacitance, *G*_leak_ and *E*_leak_ are the leak conductance and reversal potential, *E*_exc_ and *E*_inh_ are reversal potentials for excitatory and inhibitory postsynaptic currents. For each equation *i*, *i* = 1,…3, *I*_inj_*^i^* is the injected current, *V_mi_* is the membrane potential for that level of current injection. The value of *C_m_* is obtained from the time constant of an exponential fit to the membrane voltage response to a test hyperpolarizing pulse applied at rest. *G*_leak_ is the constant component (at least 50%) of the conductance observed at rest and the reversal potential *E*_leak_ is fixed at −80 mV (see details in Monier et al., 2008).

## Results

### Design of a virtual eye-movement exploration model

The aim of these experiments was to measure the temporal precision and trial-to-trial variability of V1 responses to stimuli which mimicked the visual exploration of a natural environment (Vinje and Gallant, 2000; Rucci and Desbordes, 2003) and to compare them with more classical artificial stimuli. In natural free behaving conditions the retinal image is never still. It is continuously updated, not only by saccadic but also by fixational eye-movements, including drifts, microsaccades, and tremors (see Martinez-Conde et al., 2004 for a review). In the spatial domain, we chose a natural scene of likely occurrence for a cat, i.e., another cat in a flower field (presented in Figure 1A). The spatial correlation structure of this image obeys a power law (see below), a feature characteristic of natural scenes (Ruderman and Bialek, 1994; review in Simoncelli and Olshausen, 2001). To simulate a realistic retinal flow in the anaesthetized and paralyzed preparation, we animated the natural scene along an artificial scanpath, with kinematics parametrically adjusted to eye-movement statistics measured in the behaving animal (Collewijn, 1977; Olivier et al., 1993; Figure 1B). The advantage of blocking eye-movements in the paralyzed preparation was to produce the exact same scanpath from trial to trial, a situation that cannot be achieved in the behaving animal, even when the task is to maintain target fixation (Vinje and Gallant, 2000). Note however that our paradigm does not include the proprioceptive and efferent copy extraretinal signals triggered during active behavior. The same scanning protocol was applied in all experiments independently of the characteristics of the recorded RF.

The animation of a natural scene by simulated eye-movements has a drastic effect on the spatiotemporal statistics of the luminance profile falling on one point of the retina, hence seen by a given RF. The dynamic profile of the local contrast information, as a result, strongly departs from the one classically imposed by visual neurophysiologists (DG and DN conditions in Figure 1A). The temporal power spectra of the luminance signal falling on one retinal photoreceptor (approximated here by a single image pixel) are shown for each stimulus condition in Figure 1D. For stimuli animated with eye-movements (dark curve, NI), the power spectrum presents a 1/*f*^γ^ shape, somewhere in-between the flat spectrum of the white noise (blue curve, DN) and the multiple harmonic spectrum of the DG (red curve, DG). The γ values obtained for natural scene viewing conditions are around −1.8 (−1.79 for our model), a value close to that observed for a Brownian signal. However, Figure 1 shows that measurements have to last several seconds in order to be reproducible and smooth-out non-stationarities, indicating the existence of finer temporal structures in the contrast/luminance signal.

Twenty-two cells were subjected to four sets of stimuli of increasing complexity: (a) a DG of optimal orientation, direction, and spatial and temporal frequencies, (b) the same optimal grating animated by a modeled eye-movement sequence (GEM), (c) a NI animated by the same virtual scanpath, and (d) dense binary white noise (DN). Sixteen of these cells, which were recorded long enough to apply the complete protocol (see Materials and Methods**)**, were used for the comparative analysis presented below. Six other cells were used for conductance measurements.

### Reliability, precision, and sparseness of the spiking responses

**Figure 2:**
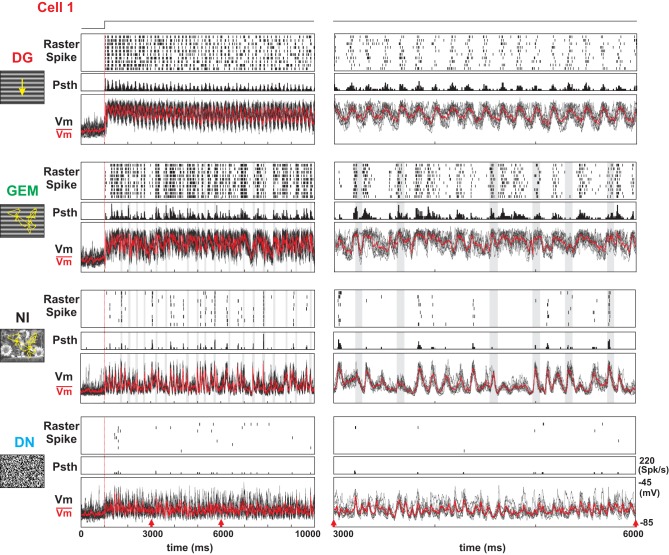
**Dynamics and Reliability of intracellular evoked responses as a function of visual input complexity**. Subthreshold intracellular (*Vm*) and spiking responses of a Simple cell to four types of full screen stimulus animations (indicated at left), at two different temporal magnifications. **Left panels**: full duration of the responses. **Right panels**: zoomed in section (3s-long duration, indicated by red arrows). From top to bottom: optimal sinusoidal luminance grating, drifting at 6 Hz (DG); same grating animated by saccadic and fixational eye-movements (GEM); natural image animated by the same sequence of eye-movements (NI); binary dense white noise (DN). For each condition, the three rows represent respectively the trial-by-trial raster of spike trains, aligned with the movie onset (vertical red bar), the PSTH, and the superimposed stimulus-locked *Vm* trajectories after spike removal of individual trials (black) and their waveform average (in red). Shaded gray stripes indicate saccade occurrence.

**Figure 3:**
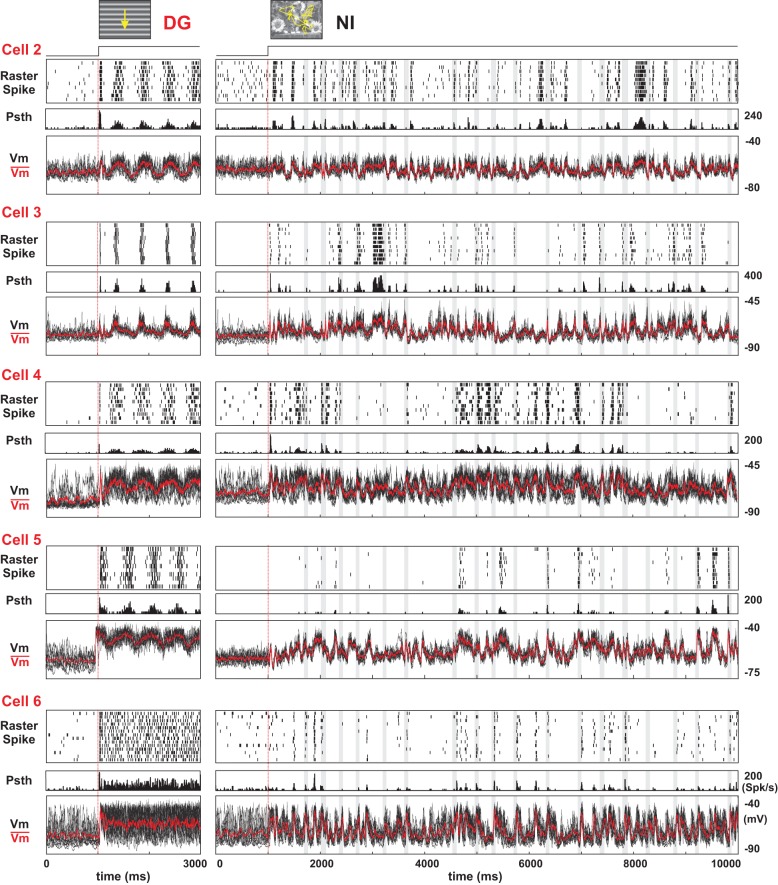
**Comparison of spiking regime and subthreshold dynamics for DG and NI**. Stimulus dependence of evoked dynamics in five other cells (labeled Cell2 to Cell6). Format is the same as in Figure 2 (see legend). In each of these cells, dense firing and high variability in spike timing across trials are observed for DG whereas sparse and reliable firing predominate for NI. Cells are ranked from top to bottom rows according to their Simpleness behavior (measured by the *F*1/*F*0 ratio of the spike rate and *Vm*). The shaded stripes indicate the occurrence of saccades.

Figure 2 illustrates the subthreshold and spiking responses (as well as averaged PSTWs and PSTHs) of a representative Simple cell to the 10 repetitions of each of four stimulus conditions. For each condition, two different response periods are shown: the onset (left panel) and the asymptotic activation regime (3 s after onset, right panel, different time-scale). The presentation of an optimal DG (top row) evoked a strong modulation of both the sub- and supra-threshold responses at the grating temporal frequency (spike *F*1/*F*0 = 1.26). As expected from previous studies, the high temporal frequency components of the response varied considerably from one trial the next. However, this trial-to-trial variability in the spiking behavior appeared stimulus dependent: it was significantly reduced by increasing the complexity of the spatio-temporal input spectrum. Animation of the same grating through simulated eye-movements (GEM, second row from the top) did not affect the mean evoked activity level but produced spiking episodes, which were highly reliable across trials. An improved level of precision was found for NI animated by eye-movements (third row from the top), with a much sparser spiking pattern (mean firing rate decreases by 74% from DG to NI). In the DN condition (bottom row) the spiking response was further reduced, and no consistent spiking response emerged from one trial to the next. The same stimulus dependence and contrasted behavior in spiking response patterns between DG (dense and variable) and NI (sparse and precise) were reproduced across the whole population of recorded cells, irrespective of the level of “Simpleness” of the recorded RF, as illustrated by five more examples in Figure 3.

**Figure 4:**
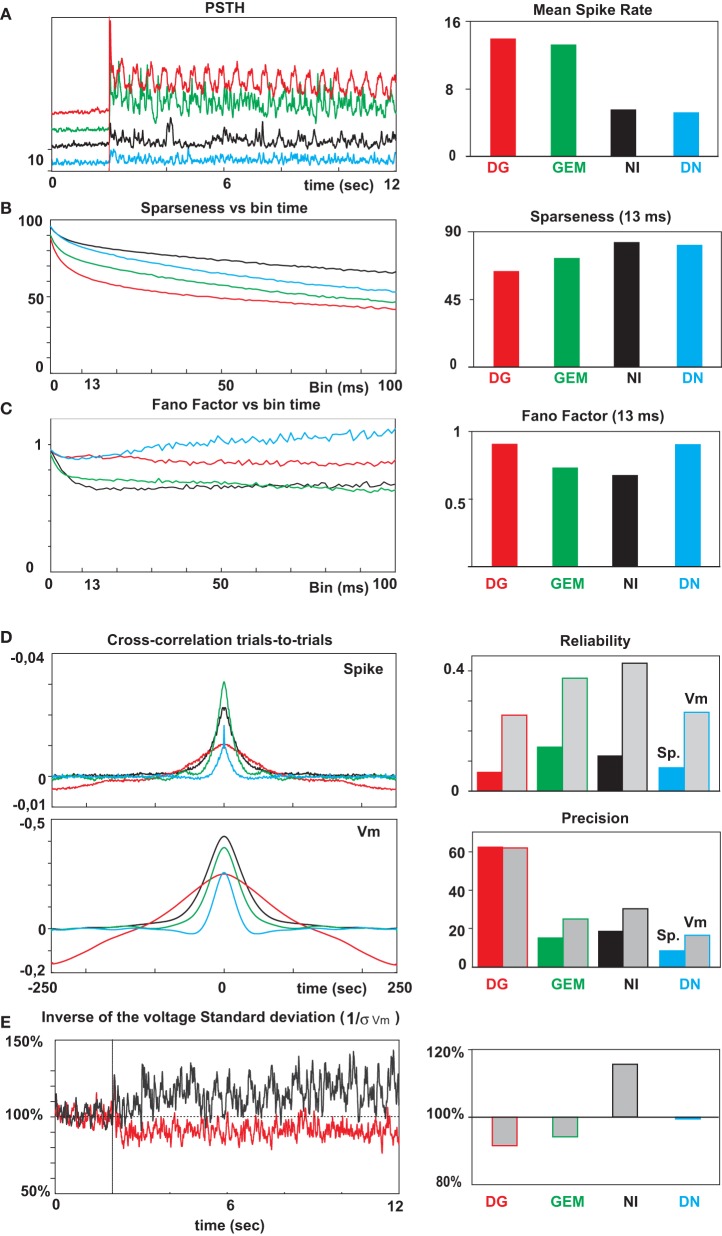
**Mean firing, reliability and temporal precision of spiking events**. From top to bottom: **(A)** Mean spike activity temporal profiles (PSTH) and mean evoked spike rates (right panel) for the each of the 4 stimulus conditions (same color code as in Figure 1). **(B)** Left, the sparseness index (Vinje and Gallant, 2000) was computed for each condition and its temporal evolution is shown for bin durations ranging from 1 to 100 ms (step of 1 ms). Right, the sparseness mean values, averaged over the whole stimulus presentation, were estimated with a temporal bin equal to twice the screen refresh rate (13.3 ms) used for the temporal animation; **(C)** Same graphs for Fano factor; **(D)** Crosscorrelation (CC) functions across trials for spikes (top) and subthreshold *Vm* activity (bottom). The reliability is given by the peak amplitude at time zero, and the temporal precision by the standard deviation of the Gaussian fit of the CC functions, expressed in ms. **(E)** Left, the reliability waveforms, estimated by the inverse of the stimulus-locked SD (1/s) across trials, were averaged across the 20 cells after normalization of the basal ongoing level for each cell separately prior to the stimulus onset. Right panel, the relative change from ongoing activity variability is expressed for each of the protocols.

In order to quantify the reliability and sparseness of the evoked discharge process at the population level and compare our results with previous studies, we performed an extensive analysis of various indexes. The main observations are summarized in Figure 4: The top row represents the mean spike activity temporal profiles (PSTH) and mean evoked spike rate (right panel) over a moving averaging window of 10 ms for the each of the 4 stimulus conditions. As seen in the cell examples, the response mean rates are higher for DG and GEM, and much lower for NI and DN. The second row shows classical measures of sparseness for bin durations ranging from 1 to 100 ms. In order to compare our results with the study of Vinje and Gallant (2002) more directly, the sparseness mean values (S index, see Methods) were averaged over the whole stimulus presentation with a bin equal to the refresh rate of stimuli (13.3 ms). This bin value is the same as used in Vinje and Gallant for their movie animation. In the third row, we illustrate the Fano factor (FF) at 13.3 ms and the FF for bin durations ranging from 1 to 100 ms. The sparseness for NI and DN stimuli are higher than for other stimuli (83.4 ± 14.8% for NI and 81.4 ± 18.8% for DN vs. 64.5 ± 18.3% for DG, 73.0 ± 17.8% for GEM).

The Fano Factor (FF) were sub-Poissonian for the animated stimulation protocols (0.73 ± 0.14 and 0.67 ± 0.10 for GEM and NI, respectively, Figure 4C). These values were significantly lower (*t*-test, *p* < 0.001) than the FF values obtained for DG and DN protocols (0.90 ± 0.20 and 0.89 ± 0.18, respectively). It should be noted that the similarity of these dispersion measurements made for the GEM and NI conditions results from correlated but opposite changes in mean and variance: when switching from GEM to NI, the mean firing rate was considerably and significantly reduced (13.0 Hz vs. 5.5 Hz on average; *p* < 0.005), but so was the variance (from 0.21 to 0.16 on average, *p* < 0.005) (Figure 4A), which resulted in similar FF values (Figure 4B). Since very low firing rates can bias the FF estimation toward higher values, we also did our estimation by restricting it for the subset of cells whose firing rate was above 5 Hz (11 cells for DG and GEM protocols; 5 cells for NI and DN protocols). In this restricted cell population, the averaged FF were respectively 0.72 for DG, 0.60 for GEM, 0.69 for DN, and 0.50 (the lowest) for NI conditions. We also checked the dependence of the measurements on the bin the size of the time integration window: the plots in Figure 4C show that the FF value for DN increased significantly and constantly (1.28 ± 0.57 at 250 ms), when the FF values for NI and DG conditions remained stable (0.71 ± 0.25 and 0.91 ± 0.40 at 250 ms). In contrast, the FF values of GEM decreased and became smaller than the NI ones for bin sizes larger than 60 ms (0.62 ± 0.18 at 250 ms). These observations show that FF was consistently sub-Poissonian for the GEM and NI stimulations, despite the low firing rate in this latter (sparse) condition. In both cases, the spiking activity remained highly irregular (respective ISI CV for GEM and NI: 1.27 ± 0.24 and 1.35 ± 0.22, 1.27 ± 0.27 and 1.34 ± 0.37 for DG and DN respectively).

We also computed the reliability and the temporal precision of the spiking response (Butts et al., 2007) by fitting a Gaussian function to the cross-correlation of the spiking response between trials. The reliability is given by the CC peak amplitude at time zero, and the temporal precision by the standard deviation of the Gaussian fit. The reliability of spiking events (Figure 4D) is much higher for GEM and NI than for the other stimuli (0.145 ± 0.086 for GEM and 0.114 ± 0.05 for NI vs. 0.062 ± 0.037 for DG and 0.079 ± 0.05 for DN). A similar effect was found for the membrane voltage except that the reliability was higher for NI (0.42 ± 0.13) than for GEM (0.37 ± 0.13). The precision of the spiking response was the highest for DN, in the range of 10 ms (9.1 ± 1.9 ms) and slightly lower, on average 10–20 ms, for NI and GEM (14.8 ± 4.4 ms and 18.2 ± 6.3 ms, respectively). These values are similar to what has been found in the LGN (Reinagel and Reid, 2000; Butts et al., 2007; Kremkow et al., submitted, using the same dynamic stimulus seed). In contrast, precision was degraded for DG protocol (62.3 ± 30.2 ms, *p* < 0.001, *t*-test), indicative of the necessity of a rate code and average across trials. The same trend is observed in the precision of membrane voltage.

From this first step analysis at the spiking level, we conclude that natural scenes are an example of sensory stimulation where a low evoked firing rate is coupled with a high spiking reliability and a high temporal precision. These results, replicated in 20 cells, suggest that the irregular subthreshold activity imposed by the full field natural scene movie induces reliable and precise spikes, despite the fact that the input statistics of NI do not necessarily optimize the discharge level (like for DG and GEM). Note here that, apart from very few cells, the majority of cells were presumably excitatory, since their firing patterns in response to current pulses were typical for regular and bursting cells. Our data indicate a rather homogeneous population in terms of visual behavior (but see Haider et al., 2010 and Hofer et al., 2011, for evidence of different stimulus statistics dependence between excitatory neurons and inhibitory interneurons). We also emphasize the fact that similarly high levels of spike reliability are obtained in two different ways in NI and GEM conditions, since the mean and variance are lowered by a same scaling factor in NI when compared to GEM. If DN remains the condition where spike timing is the most precise, both reliability and mean activities are very much on the low side. Depending on the cell, stimulus efficiency may vary dramatically during DN, from one trial to the next. In fine, this comparative study allows us to state, on a cell-by-cell basis, that NI is the only condition which maximizes the temporal precision and reliability of a sparse code.

### Reproducibility in subthreshold membrane potential dynamics

Intracellular recordings give simultaneous access to both the spike pattern and the somatic echoes of the synaptic input bombardment. For the preferred grating (DG), Simple cells exhibit a periodically modulated dense spiking activity, whereas the reliability expressed across trials was low, both for spike timing and response strength (cell 1 in Figure 2 and cells 2–4 in Figure 3). At the subthreshold level, a periodic modulation of the membrane potential was detectable (as classically reported for Simple cells) and followed the low temporal frequency of the drifting grating. A high level of variability in *Vm* time-courses was seen across trials, as best illustrated in the expanded timescale panels (individual trials in black, mean waveform in red in the top row of Figure 2; see also left panel in Figure 3). In contrast, the NI raster plots for the same cells show spiking events occurring at precise times/delays during the movie clip (third row from top in Figure 2, right panel in Figure 3). The intracellular *Vm* records show in addition that, for NI conditions, the precise postsynaptic spikes were riding on reliable, fast and temporally structured subthreshold fluctuations (Figures 2, 3). Importantly, the fast components of the membrane potential trajectory showed a high trial-to-trial reproducibility, even when the cell was not firing, for silent periods extending for several hundred of ms prior and after the reliable spiking event (see also the central panel in Figure 6). The responses to NI seemed thus to result from irregular yet reliable network activity. The GEM response was also irregular and reliable, but gave rise to many more spikes (second row from top in Figure 2). Finally, in the DN case, the fast *Vm* fluctuations were reliable, but with a relatively lower occurrence of slow depolarizing events than observed in the NI and GEM conditions, and consequently resulted in a much lesser efficiency in spike generation (bottom row in Figure 2). In many neurons, the reduced amplitude of evoked *Vm* fluctuations for low frequencies (below 10 Hz) was such that these cells seldom reached the spiking threshold (Figure 6, right panel). However, this latter effect was not present in two biocytin-labeled Simple cells, identified in layer 4 and 6 and probably receiving a direct input from thalamus [see layer 6 cell (Cell 7) example, Figure 12, right column].

A reliability and precision analysis was carried out at the *Vm* level by comparing waveform correlation across trials, and the conclusions support a stimulus context dependence similar to that revealed by the analysis of spike rasters (Figure 4). More unexpectedly, noise levels observed in the voltage dynamics strongly differed across stimulus conditions. The observation of a reduced level of stimulus-locked variance, induced from the stimulus onset and maintained during the whole stimulus presentation, can be seen at the population level: the reliability increases during NI, (+15.5%) and conversely decreases for DG (−11%; see the overlaid 1/σ waveforms in the bottom row in Figure 4). However, this method remains very sensitive to fluctuations in the high frequency range (>100 Hz), and other frequency-dependent analysis methods had to be developed by trying to differentiate between Signal and Noise, as detailed below.

**Figure 5:**
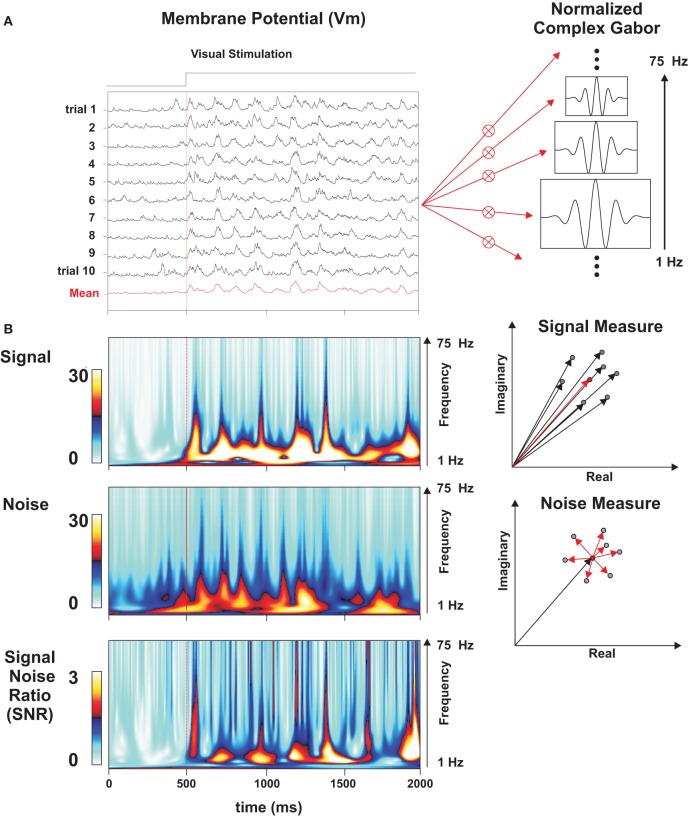
**Wavelet analysis and time-frequency estimation of SNR. (A)** Rasters of *Vm* subthreshold responses for a set of individual trials in a Simple cell (*F*1/*F*0 = 1.84, different from that shown in Figure 2) to a grating animated by virtual eye-movements (GEM condition). On the right, schematic representation of an array of Gabor wavelets ranging from 1 to 75 Hz. **(B)** Time-frequency analysis of the evoked Signal (upper matrix), the Noise (middle), and the SNR (bottom matrix), following the method of Croner et al. (1993). The repetition of the vectorial operations (detailed in the right panels) at all times and frequencies yields the Signal and Noise matrices. The SNR matrix is obtained from point-by-point division of the Signal matrix by the Noise matrix. Reliable events are signaled by hot (red) peaks straddling from low to high frequencies (1–75 Hz). Upper right panel: each black vector represents the result (in the complex plane) of the convolution of the signal with a given wavelet frequency for one particular point in time and a given trial. The red vector represents the mean vector, averaged across all trials, and its squared modulus gives the estimated Signal power. Lower right panel: Noise is measured in the complex plane as the average distance (dispersion) of the individual trial vectors (black vectors) from the mean (red).

**Figure 6:**
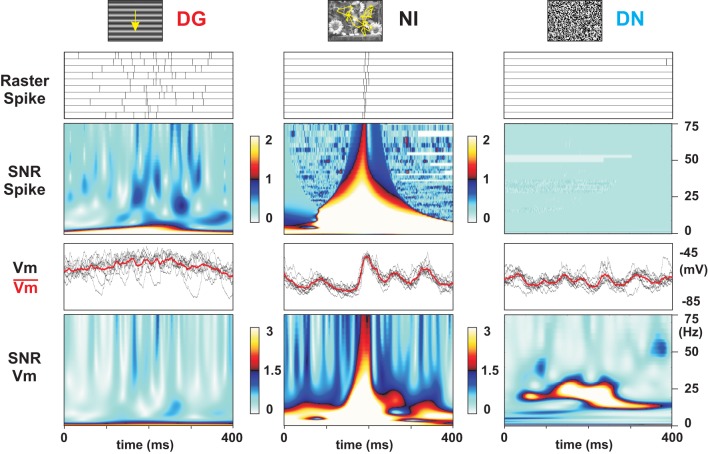
**Stimulus-dependent reliability of spiking and *Vm* responses**. Comparison of time-expanded epochs of the responses of another Simple cell to an optimal grating drifting at 2 Hz (DG, left column), a NI animated with eye-movements (middle column) and DN stimulation (right column). From top to bottom: (i) raster and frequency-time SNR analysis of the spiking responses; (ii): superimposed individual trial waveforms of the subthreshold *Vm* responses and SNR analysis. The chosen 400 ms epochs (starting at the same latency from stimulus onset for a given stimulus condition) illustrate the periods of strongest spiking (DG and NI) and subthreshold (DN) activation for the same cell. As generally reported, dense spiking and highly variable *Vm* trajectories were observed during the optimal phase of the grating (DG). In contrast, the same cell exhibited, for each trial, a highly reliable burst of activation at the spiking level (1–3 spikes with a 5–10 ms precision). Note that the *Vm* trajectory was almost noiseless several hundred ms before and after the spiking event(s). The subthreshold behavior in the DN condition was also more reliable than for DG. However, the beta-range activation observed for DN lacked the low frequency power necessary to reach spike initiation.

**Figure 7:**
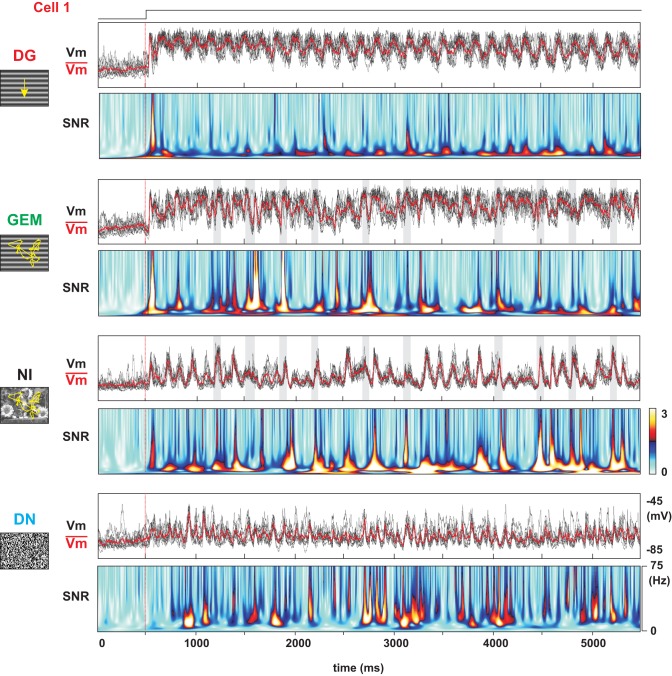
**SNR analysis of the stimulus-dependent reliability of the pre-synaptic bombardment**. From top to bottom, subthreshold *Vm* dynamics and SNR power matrix in another Simple cell for the four stimulus conditions, DG, GEM, NI, and DN. Each panel represents 500 ms of ongoing activity followed by 5 s of continuous visual activation. Note the colored peaks (in the SNR time-frequency matrix) signaling highly temporally structured input in the NI and DN conditions. In the DG condition, the only reliable event is the quenching observed a few tens of ms after at the stimulus onset (red vertical line at 400 ms). The shaded stripes indicate the occurrence of each saccade.

**Figure 8:**
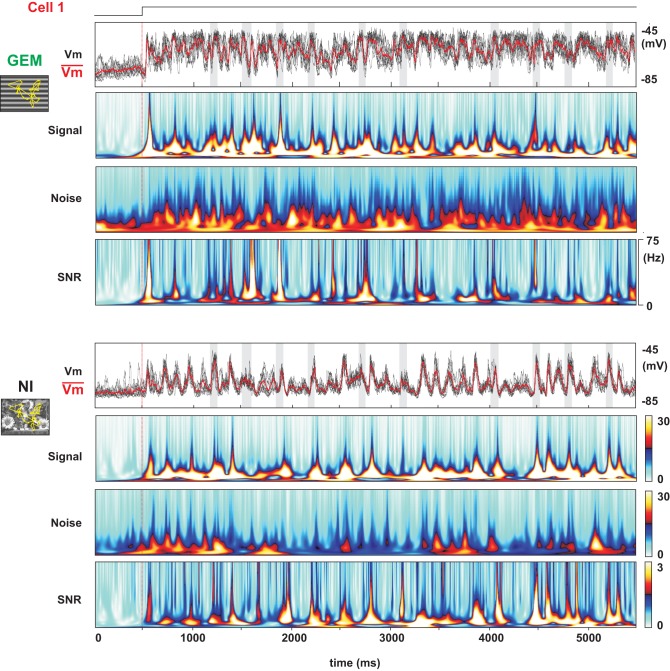
**Detailed Signal-Noise analysis of the stimulus-dependent reliability of the presynaptic bombardment**. Same cell as in Figure 7 for two stimulus conditions (GEM, NI). The upper traces represent the individual trial (black) and mean (red) stimulus-locked waveforms. From top to bottom: Signal, Noise and SNR time-frequency matrices. In spite of the similarity in the SNR patterns, note that the optimization of the SNR ratio results from a stronger Signal in the GEM condition, and a reduction of Noise in the NI condition, compared to the DG condition (see Figure 7).

Since the reproducibility of the membrane potential trajectory for each protocol may depend on the timescale chosen for the response analysis, we quantified the temporal evolution of the trial-by-trial reproducibility by performing a time-frequency wavelet analysis of both the spike and *Vm* responses. The method (illustrated in Figure 5) can be viewed as an extension of the signal and noise estimation proposed by Croner and colleagues to the time-frequency domain (Croner et al., 1993). Each of the ten individual trial-responses to a given stimulus was filtered by an array of complex Gabor wavelets whose temporal frequencies ranged from 1 to 75 Hz. Thus, a set of ten complex numbers (one for each trial of the same stimulus) was computed for each frequency band and point in time. The mean (the Signal) and standard deviation (the Noise) in the complex plane were used to build SNR matrices (Figures 5–8). This decomposition allows the extraction of several time-frequency dependent measures: Signal power, Noise power, and SNR, as illustrated in Figures 7, 8 (same cell as in Figure 2).

**Figure 9:**
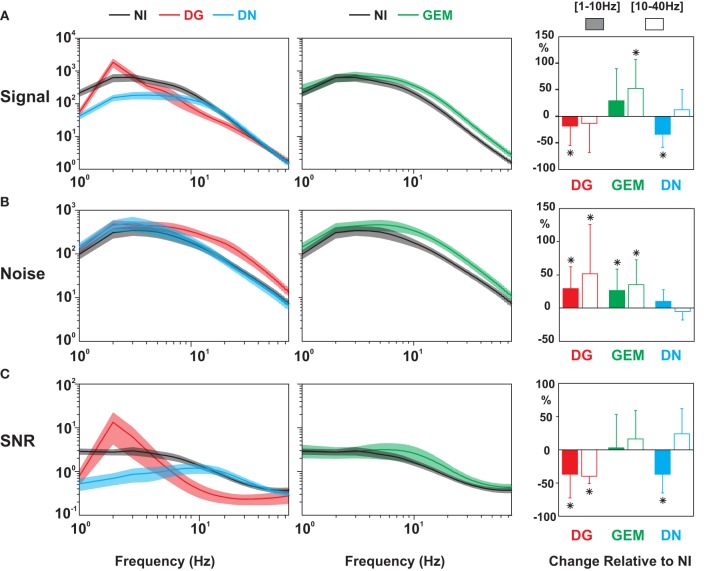
**Population analysis of the temporal power spectrum, for Signal, Noise, and Signal-to-Noise Ratio (SNR)**. Comparison of the average temporal power spectra of the Signal (top row), the Noise (middle) and the SNR (bottom) across protocols. For clarity, NI spectra (black) are displayed twice, on the left with DG (red) and DN (blue), and on the right with GEM (green) conditions. Shaded areas indicate ± 1 s.e.m. Same ordinates and temporal scales for **(A–C)**. The right column represents the relative change in the power density for specific bands of temporal frequencies [(1–10 Hz) (shaded box) and (10–40 Hz) (empty box)] for three stimulus conditions (DG in red; GEM in green, DN in blue) relative to the NI case. Stars indicate a significant statistical difference with the NI condition (Wilcoxon paired test, *p* < 0.05). Note again that the broadband reconfiguration of the SNR spectrum in the NI and GEM conditions toward higher frequencies results from two different processes (Signal power increase for GEM, and Noise decrease for NI).

The SNR measure captures transient and reproducible fluctuations which appear as “hot peaks” in the corresponding SNR matrices. When associated with a reliable spiking event (see for instance NI condition in Figures 6–8), these peaks straddle from low to high temporal frequencies. When applied to epochs where the cell was not firing, the same analysis detects highly reliable *Vm* responses: in the NI condition, for instance, hot SNR bands are visualized in the frequency-time matrix in the β − γ temporal frequency range (15–60 Hz) (Figure 6, middle panel and Figure 7, third row). These observations contrast with the low temporal precision of the responses to DG: the trial-to-trial reliability of high temporal frequencies in both the *Vm* and the spike responses was low, and as a consequence, high SNR values were restricted to a band corresponding to the driving temporal frequency of the drifting grating (Figure 6, left panel and Figure 7, top panel). In the DN condition, reliability in the high temporal frequency components of the *Vm* was often detected, specifically in the β range. However, there was a marked absence of low temporal frequency components in this condition, which seems correlated with a lack of slow depolarizing voltage events crossing the spike threshold (Figure 6, right panel and Figure 7, bottom panel). These differences between stimulus conditions were further quantified by averaging the SNR values for each frequency band over the whole duration of the movie. This gives a SNR spectrum for each protocol and each cell. The left panel of Figure 9C shows the averaged SNR spectra over the population of all recorded cells, for the 3 protocols: DG, NI, DN. The NI averaged spectrum is significantly higher than the DG spectrum for high frequencies (above 20 Hz, *p* < 0.005), while it is significantly higher than the DN spectrum for low frequencies (below 10 Hz, *p* < 0.06).

### Differential contextual dependence of signal and noise

A diversity of stimulus-dependent mechanisms is further revealed when decomposing the SNR of the subthreshold *Vm* activity in its Signal and Noise components. In the DG case (not illustrated), Signal power is high at the drifting temporal frequency (which can be expected from a “Simple” cell following the driving frequency), while the Noise power is high over the whole spectrum. This results in a low SNR, except at the drifting frequency (Figure 6, left panel and Figure 7, top panel). In the DN case, the SNR is high in the medium and high temporal frequency range, in agreement with the hot bands observed in the β frequency domain of the SNR matrices (Figure 6, right panel and Figure 7, bottom panel). This is due to the fact that the Noise spectrum is low over a broad range of frequencies (similarly to the NI condition), but that the Signal power is high only for frequencies above 10 Hz. This latter effect may be expected since, by definition, the DN stimulus spectrum is “white,” and there is less power in the DN stimulus in the lower frequency band than in the other ones. The absence of large depolarizing events in the DN-evoked response results in a low, unreliable spiking behavior in cortical neurons which are not the direct of target of thalamo-cortical afferents. The finding that large *Vm* SNR at high frequencies does not guarantee a reliable spiking is reminiscent of the demonstration already made *in vitro* that a broadband somatic current signal is required, covering both *low* and *high* frequencies, in order to produce a reliable spiking activity (Mainen and Sejnowski, 1995; Nowak et al., 1997). It has also been shown *in vivo* that spikes are more reliable when fast depolarization's ride on a slower depolarizing wave, because of a lowered spike threshold (Azouz and Gray, 2000).

Compared to the DG and DN protocols, the NI stimulus has the particularity to enhance the SNR over a broad range of frequencies. By comparing the responses to NI and GEM stimuli, we examined if this SNR enhancement was specific to the NI statistics. The Signal and Noise decomposition for these conditions is shown in Figure 8. The GEM stimulus is animated by the same temporal sequence of eye-movements as in NI conditions, but the spatial content of the natural image is replaced by a grating optimized in orientation for the recorded cell. The GEM and NI conditions evoked similar SNR, and this over a broad range of frequencies (Figure 8). In the presented cell (Cell 1, same as in Figure 7), as in many others, although the distinctive peaks in the SNR frequency-time matrix show—both for the NI and GEM conditions—the presence of highly synchronized epochs of high SNR straddling across all frequencies, the NI movie onset seems to recruit a contextual Noise level much lower than the GEM stimulus.

The estimation of the SNR spectrum over the whole population of cells (Figure 9C) confirms that the SNR levels for the GEM and NI conditions are comparable over a broad range of frequencies (*p* > 0.20 for frequencies between 1 and 75 Hz). The NI is thus not the only stimulus evoking a high SNR over all frequencies. We then compared—between NI and GEM conditions—the origin of this high SNR by estimating the Signal and Noise components (Figures 9A,B, middle panels). *On one hand*, as expected, the power of the Signal was higher in the case of the GEM stimulus (*p* < 0.01) between 15 and 60 Hz (relative change with NI plotted in the right panel of Figure 9A). *On the other hand*, the contextual Noise spectrum power was lower in the NI condition (middle pane in Figure 9B; see also the frequency-time Noise Matrix in Figure 8). This effect of Noise reduction was particularly striking at high frequencies (right panel in Figure 9B, 10–40 Hz (*p* < 0.01)****. Thus the opposed changes of Signal and Noise of the *Vm* dynamics across the two stimulation contexts result paradoxically in comparable SNR values at the *Vm* level (Figure 9C). As shown above, they gave rise in both cases to reliable spiking responses, although the mean firing rate was much higher for the GEM responses.

We conclude from this analysis that the statistical properties of the spiking responses detailed in the first section of the *Results* reflect the differential contextual effects on Signal and Noise revealed at the subthreshold level: similar spike-based FF values are obtained for GEM and NI, but with different mean firing rates, resulting from distinct Signal and Noise modulations at the subthreshold level. When taken together, our results exclude a univariate (or multiplicative) relationship between Signal and Noise in the subthreshold *Vm* activity: depending on the visual stimulus, a decrease in Signal can be accompanied by either a decrease (from GEM to NI) or an increase (from GEM to DG) of the contextual Noise component. Thus, the feature-dependent selectivity of the Signal does not necessarily match that of the Noise. The latter seems to decrease at the synaptic level, especially when the complexity of the stimulus increases, reaching minimal values for NI and DN.

### Impact of the retinal flow induced by virtual eye-movements

**Figure 10:**
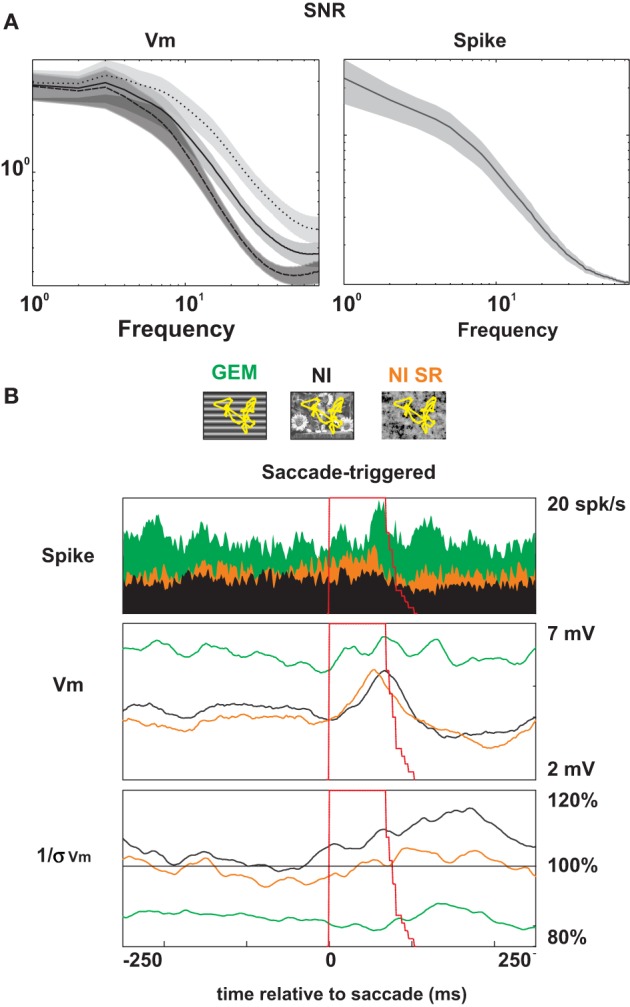
**Population saccade triggered analysis. (A)** SNR spectra of the *Vm* (left) and the spike (right) responses to NI, averaged over saccadic periods (dotted line), fixation periods (dashed line) and the whole presentation (solid line). Shaded areas represent ± 1 s.e.m. Note that the 3 curves are superimposed in the case of the SNR spike. **(B)** Saccade-triggered averages of the Spike discharge (filled PSTHs in the upper panel), the membrane voltage (middle, *Vm*) and the inverse of the stimulus-locked standard deviation [right, (1/s [*Vm*])] responses. The saccade duration distribution tail (dashed red line) is overlaid with these graphs and shown as a stacking of intervals with the same onset, ranging from 70 to 110 ms duration. Three stimulus animation conditions (with saccades) are compared: grating with eye movements (green, GEM), natural image with eye-movements (black, NI) and natural image with eye movements where the spatial phase has been randomized (orange, NI-SR).

The scanpath was a stereotyped eye movement sequence which alternates large saccades with fixational eye-movements (drifts, tremor…). We wanted to distinguish the contributions of these different perturbations of the retinal image in shaping reproducible responses. More precisely, we asked whether the fast, transient displacement flow induced by saccades was the only way to elicit temporally precise and reliable responses. Results show that the reliable epochs detectable across trials at the subthreshold as well as the spike level were not strongly correlated with saccades. They also occurred during fixations (third row from the top in Figure 2; cells 2, 3, and 5 in Figures 3, 6). Furthermore, saccades did not always evoke precise responses [shown by shaded striped periods in the right columns of Figures 2 (for GEM and NI), and Figure 3 (for NI)]. We analyzed the role of saccades by segmenting the SNR matrices for the NI induced responses into two concatenated parts: the saccadic periods (starting at the onset and terminating 100 ms after the end of each saccade), and the fixation and glissade periods (complementary of the saccadic periods) (Figure 10). We compared the mean SNR spectra of the subthreshold activity (integrated over the whole duration of the response) during (dotted curve) and outside (dashed curve) saccade periods. The spectrum amplitude of the subthreshold signal was higher for saccadic periods than outside them, with a significant difference (*p* < 0.001) for frequencies between 10 and 75 Hz (Figure 10A, left). However, it has to be noted that the SNR spectrum remained high even during the fixation periods (for example, between 8 and 25 Hz, it was found significantly higher than for DG conditions (*p* < 0.02), not shown). In contrast, for spiking activity, the SNR was undistinguishable between saccadic and the non-saccadic periods (Figure 10A, right), and there was, at the population level, no significant difference between the firing rates (PSTHs) during or just after the saccades and during fixation. An illustration can be found in the expanded time scale panel in Figure 2, where precise spiking is observed repetitively in successive fixation periods, clearly outside the postsaccadic rebounds.

This conclusion is reinforced by the comparison, shown in Figure 10B, between the saccade-triggered voltage waveforms (PSTW of the mean and standard deviation in two lower panels), and the saccade-triggered discharge (PSTH, filled colors in the upper panel): in the GEM condition (green color code), the firing increases around the end of the saccade whereas variable effects across cells are seen at the *Vm* level (some cells modulating with the saccades, others not). In contrast in the NI condition (black color code), the mean firing rate (PSTH upper panel in Figure 10B) was generally unaffected during the saccade whereas a slow depolarizing bump was systematically noticeable at the subthreshold *Vm* level just before or at the terminal phase of the saccade. This differential effect in postsynaptic integration, suggestive of an elementary form of saccadic invariance, did not seem to be linked to the spatial structure of the stimulus since there was also no significant difference between the PSTWs and SNRs measured at the *Vm* level during NI and spatially randomized NI-SR (orange color code) conditions.

However it should be noted that a few cells fired more during the saccade than during fixation, while others showed the opposite trend, suggestive of some diversity across cells sensitivity to eye-movement dynamics. Interestingly, for all cells, the behaviors in GEM and NI were strongly correlated: the few cells that fired more during saccades than during fixations in NI also preferred saccades during GEM, and vice versa.

A last issue is to understand to which degree eye-movements, and in particular large saccades, participate to the contextual noise effect. Saccade-triggered reproducibility of *Vm* trajectories was compared across stimulus conditions: we found that the reduced variability observed for the NI stimulus depended on the spatial phase (compare NI and NI-SR in the bottom panel of Figure 10B) and on a 300 ms window following the saccade onset (Figure 10B, black waveform in bottom panel). However, as shown earlier (Figure 4), no strong predictive relationship could be established between the subthreshold noise level, the temporal spiking precision and specific eye-movement phases (saccades vs. fixation periods). We conclude that the saccadic-induced motion is not the only factor responsible for high SNR, and that the statistical features of fixation eye-movements are also contributing to the observed effects.

### Stimulus statistics randomization

To better understand how the statistics of the visual input affect the reliability of the responses, we presented three additional stimuli in a subset of cells (*n* = 11). We used versions of the NI stimulus for which the phases of the Fourier transform were randomized in space, time or space and time. For the spatially randomized stimulus (NI-SR), we shuffled the phases of the Fourier transform of the NI, obtained a randomized image, and animated it with the same sequence of eye-movements. For the temporally randomized stimulus, we kept the original NI, but shuffled the Fourier transform of the temporal sequence of eye-movements. Finally, we mixed both shuffling processes for the spatially and temporally randomized stimulus. In spite of significant changes in the temporal organization of spiking patterns in comparison with the NI condition, the global sparseness and timing precision of spiking events were not affected. Furthermore, these three stimuli gave levels of SNR and Signal comparable to the original NI stimulus (data not shown). Although we cannot exclude that a significant difference would appear had we recorded a larger number of cells, this result emphasizes the dominant role of the 2nd order statistics (as contained in the stimulus power spectrum) in shaping these reliable responses.

### Evidence for dynamic nonlinearities

**Figure 11:**
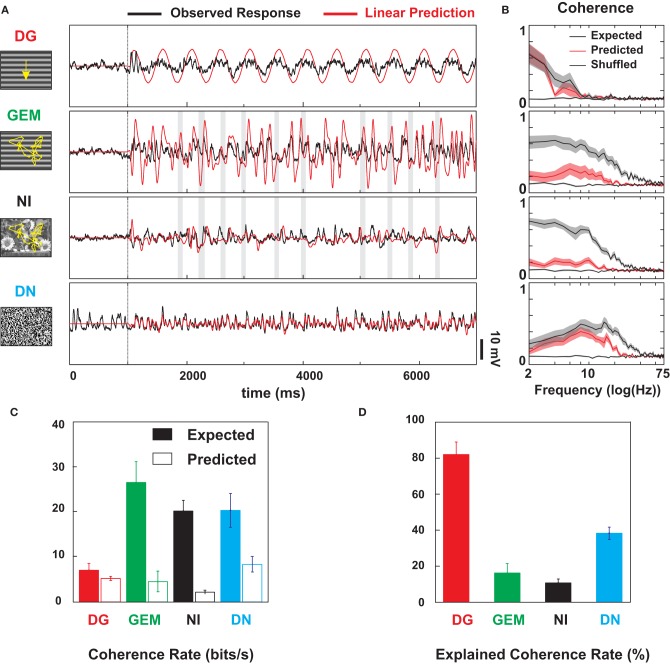
**Linear Prediction and Coherence analysis of *Vm* dynamics. (A)** Failure of the linear RF model (established with DN) to predict subthreshold membrane potential dynamics evoked by natural (NI) and eye-movement induced retinal flow (GEM) statistics. Comparison in a Simple cell of the observed (black) and linearly predicted (red) *Vm* responses, for the 4 types of stimuli (DG, GEM, NI, and DN). Linear predictions are derived from a separate set of DN stimuli used to map the recorded subthreshold RF. Note that the DN predictor accounts reasonably well for a novel DN sequence (*r* = 0.67), and for DG response, apart from a change in amplitude (divisive normalization) and temporal phase shift. In contrast, the correlation drops below significance for the NI condition (<0.3). For GEM, the prediction holds partially in the low temporal frequency domain, but the global Pearson coefficient is low (*r* = 0.42). **(B)** Population analysis (*n* = 9). Predicted (red) and expected (gray) coherence spectra (see MATERIALS AND METHODS) for each stimulus condition corresponding to same row in **(A)**. The shuffled coherence (dotted) is the baseline obtained from shuffled data. The ± s.e.m. are represented par shaded areas around the mean profile of each spectrum. Note that the non-linear behavior (the area between the gray and the red envelopes) is expressed mostly for NI and GEM, and extends over a broad range of frequencies, up to 50 Hz. **(C)** Expected (white bars) and predicted (black bars) coherence rates across protocols. Coherence rates are computed from the coherence spectra of Figure 10. The baseline coherence rate corresponding to the shuffled coherence has been subtracted. **(D)** Explained coherence rates across protocols, obtained from the ratio of the predicted to the expected coherence rates. These different panels illustrate the failure of the linear receptive field kernel to account for the subthreshold dynamics observed with eye-movements animated stimuli (NI and GEM).

Next we asked whether the stimulus dependence of the *Vm* signal dynamics resulted simply from a linear filtering of the different stimulus statistics by the RF, or involved the recruitment of non-linearities selective to natural scene and eye-movement statistics. The first-order kernel estimate was derived from the subthreshold responses evoked during an initial exploration of the RF with DN. It was then convolved with the different stimulus movies in order to obtain linear predictions of the *Vm* responses (Figure 11A). The comparison with the recorded traces shows that V1 non-linearities affect most strongly the responses to non-stationary stimuli. The temporal profile of the stimulus-locked *Vm* trajectories were drastically reshaped in the more natural-like conditions animated with virtual eye-movements (NI and GEM), whereas only the amplitude and latency of the trajectories were affected in the DG and DN conditions. Consequently, independently of changes in static gain, the peak value of the normalized cross-correlation between measured and predicted *Vm* waveforms, averaged across cells, was much lower for natural stimuli input statistics than for the DG and DN conditions (average peak correlation: 0.35 for NI and 0.36 for GEM vs. 0.77 for DG and 0.63 for DN; Wilcoxon paired test, *p* < 0.001). Another mismatch with the linear model concerns the relative amplitude of the predicted and the recorded *Vm* traces (independently of the phase). The observed amplitude of the responses to DG and GEM were systematically smaller than their linear predictions. In each condition, a divisive static gain was required to optimize the fit between the observed and predicted waveform (by minimizing the sum of the least mean square errors), ranging from 1.5 to 3.0 for DG and from 1.2 to 10 for GEM. This functional rescaling is compatible with a divisive normalization (Heeger, 1992). It agrees with other intracellular measurements from our lab comparing sparse and ternary dense noise (Fournier et al., 2011). Possible mechanisms include the non-linearity of synaptic integration (and membrane equation), intracortical shunting inhibition (Borg-Graham et al., 1998; review in Monier et al., 2008), without excluding synaptic depression (Carandini and Ferster, 2000; Boudreau and Ferster, 2005; Reig et al., 2006). We will discuss in section 8, at a more mechanistic level, the role of the temporal interplay of excitatory and inhibitory conductances and their peak amplitudes relative to the rest conductance.

At the population level, we found that the responses to full-field stimuli animated by natural eye-movements (GEM and NI) were not only more reliable, but also more non-linear than their DG and DN counterparts. To quantify this difference, we measured the linearly predicted, expected and shuffled coherences (Haag and Borst, 1997; van Hateren and Snippe, 2001). The coherence between two signals quantifies, in the Fourier domain, their degree of linear dependency: it is equal to 1.0 at each frequency when the two signals are linearly related, and is less than 1.0 when the signals are non-linearly-related and/or corrupted by noise. In each visual condition, the response reliability and non-linearity were quantified by the expected coherence (Cohexp, computed between each trial-response and the mean averaged from the other trials, black curves in Figure 11B) and the predicted coherence (Cohpred, computed between the trial-responses and their linear prediction, red curves in Figure 11B), respectively. We also computed the shuffled coherence (between time-shifted trial responses and the mean *Vm*, dashed black curves in Figure 11B), a theoretical minimum given the limited number of trials.

The across-cell-averages of the Coh_Exp_ in each visual condition (Figure 11B, black curves) are in agreement with the SNR shown in Figures 6–8 and summarized in Figure 9. Eye-movements-animated stimuli (GEM and NI) evoked reliable responses from low to high temporal frequencies (from 2 to >40 Hz), whereas the other two stimuli restricted reliability to narrower frequency ranges: low frequencies (<10 Hz) for DG and medium and high frequencies (10–40 Hz) for DN. As expected, since the Coh_Pred_ is not sensitive to the static gain factor and temporal phase shift potentially introduced in the DG response by contrast normalization (Heeger, 1992), the DG response was largely predicted by the linear RF. The DN response was mostly linear too, but one should note that significant non-linearities are expressed at frequencies above 10 Hz. On the contrary, the eye-movements-animated stimuli evoked strongly non-linear responses. Their predicted coherences were uniformly low, closer to the shuffled than to the expected coherence. These findings are summarized in Figure 11C by integrating the coherence from 2 to 75 Hz, to yield coherence rates (van Hateren and Snippe, 2001, see Materials and Methods). As shown in Figure 11D, linear mechanisms accounted for 81% of the expected coherence rate in the DG condition, 38% in the DN condition, 16% in the GEM condition and 11% in the NI condition.

We conclude that the *Vm* dynamics evoked by GEM and NI are not simply linear transforms of the eye-movements animated stimuli: rather, eye-movement statistics appear particularly apt at recruiting V1 non-linearities, in a fashion which favors the expression of temporally rich and reproducible responses.

### Conductance interplay and dynamic control of a temporal “spiking opportunity window”

The frequency-time analysis of the *Vm* responses and the dense occurrence of temporally precise SNR peaks in the afferent bombardment exclude the possibility that the sparse spiking response observed in the NI condition is generated by the convergence of only a few sparse inputs. Rather, since the SNR of the subthreshold activity is maintained at a high level over a broad range of temporal frequencies (up to 60 Hz), well-beyond the classical cut-off of the spike-based temporal frequency tuning (around 12 Hz in V1 neurons) (Movshon et al., 1978; Holub and Morton-Gibson, 1981; DeAngelis et al., 1993), our results indicate that the synaptic input bombardment for the NI condition is dense and temporally structured even when there is no postsynaptic spike (see SNR matrixes for *Vm* in Figures 7, 8). The resulting average low firing rate implies that a balanced high conductance regime is reached where intracortical inhibition competes with the barrage of excitatory events, maintaining the membrane potential below the spike initiation threshold most of the time. It is only at certain transient instances, occurring at fixed latencies during the movie presentation, that small and time-locked changes in the excitation-inhibition balance become efficient enough to create an “opportunity window” and elicit a reliable spike.

**Figure 12:**
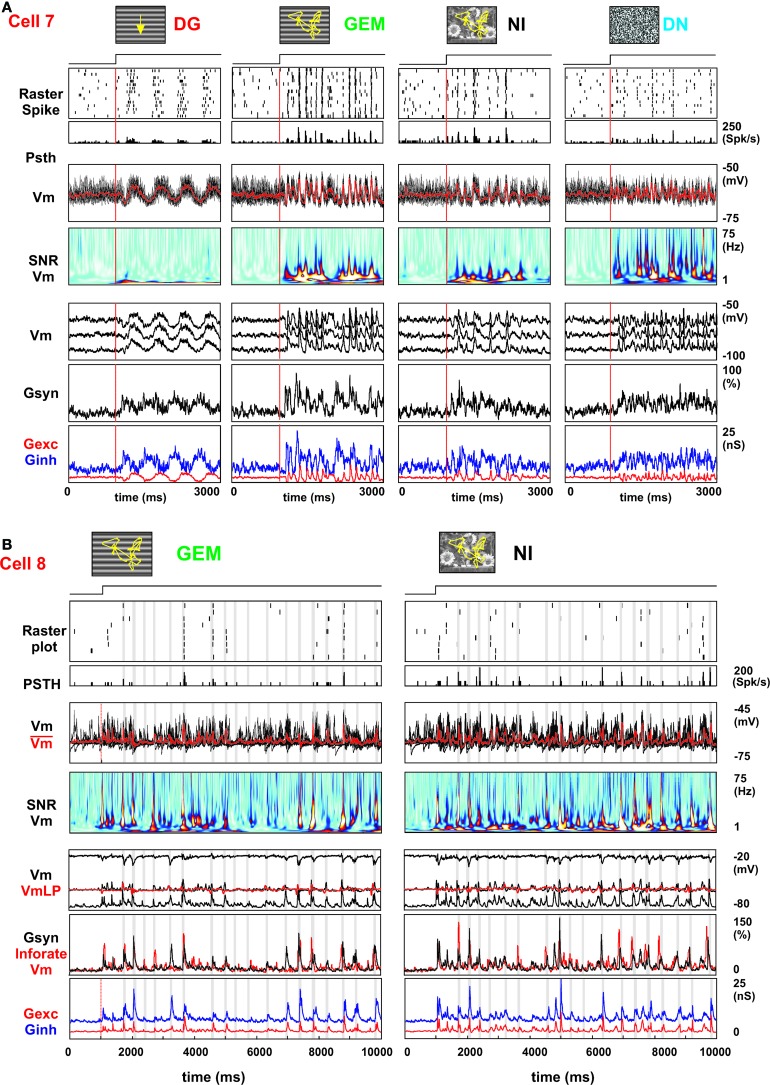
**Conductance analysis and temporal interplay between visually driven excitation and inhibition**. The continuous conductance measurements were realized in current clamp, during 10 trials of 10 s of activity each, for each stimulus and clamp conditions. **(A)** We illustrate here in a Simple Cell (cell 7), for four stimulus conditions (columns), 1 s of ongoing activity followed by 2 s of visual activity. From top to bottom, the seven rows represent the following measures synchronized with movie onset: (i) trial-by-trial raster of spike trains; (ii) average PSTH with a 3 ms time bin; (iii) superimposed *Vm* trajectories after spike removal for individual trials (black) and the mean (red); (iv) time-frequency wavelet matrix of the spike pattern SNR; (v) time-frequency wavelet matrix of the SNR of the subthreshold (*Vm*) activity; (vi) mean *Vm* trajectories average after spike removal, at three levels of current clamp [0 pA (−67 mV), −200 pA (−78 mV) and −500 pA (−91 mV)], (vii) input synaptic conductance (Gsyn in black) and (viii) reconstructed excitatory (Gexc in red) and inhibitory (Ginh in blue) conductance waveforms. **(B)** Continuous conductance measurements in a Complex cell (Cell 8) are shown for two stimuli conditions—GEM (left) and NI (right). From top to bottom: (i) raster of spiking activity, (ii) PSTH averaged across trials; iii) superimposed subthreshold activity waveforms for individual trials (black) and mean (red); (iv) time-frequency matrix of the SNR for the subthreshold activity; (v) linear predictor of the voltage waveform at 0 pA (VmLP, red), with *Vm* responses for three current levels (0 pA for control recordings, +500 pA to induce sodium channel inactivation and block spike initiation, and −250 pA), (vi) input synaptic conductance (Gsyn) and information rate of the membrane potential (InfoRate *Vm*, given by the integration of SNR[*Vm*] values over all frequencies); (vii) excitatory (red) and inhibitory conductance (blue) waveforms, synchronized with movie onset. Note that this particular cell responded briskly at each saccade occurrence (shaded vertical stripe). Same conventions as in Figure 12.

**Figure 13:**
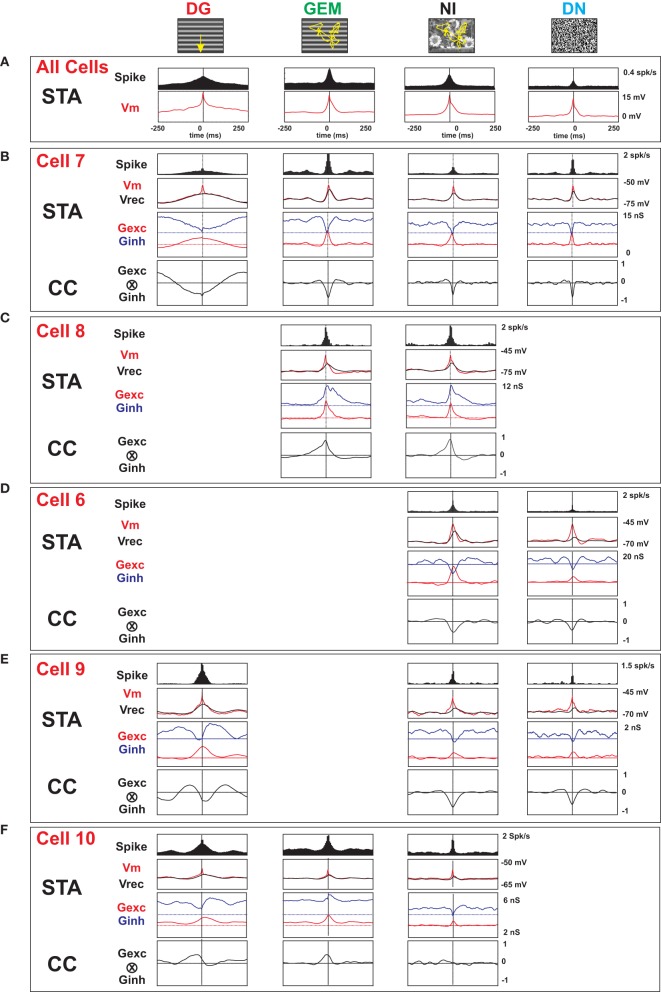
**Conductance analysis and temporal window of “spiking opportunity.” (A)** Spike trigger averaging (STA) of the spiking activity and membrane potential (*Vm*) for all the cells (*n* = 20) for all the stimulus conditions (DG, GEM, NI, and DN). **(B)** Simple Cell (cell 7), same as presented in Figure 12A. From top to bottom, spike trigger averaging (STA) of the spiking activity, *Vm* response (red), reconstructed *Vm* with conductance measurement (in black), the excitatory (Gexc, in red) and inhibitory (Ginh, in blue) conductances. The horizontal red and blue dotted lines represent the mean estimates of each conductance component at rest (Gexc and Ginh, respectively), for the four stimuli conditions. Bottom, cross-correlogram (CC) between excitatory and inhibitory conductances during all the stimulation periods, for four stimuli conditions. Note the elevated tonic Ginh level in the three visual conditions (“high conductance” state). The stimulus dependence patterns of the STA plots suggest a temporal refinement of the anticorrelation between the peak of Gexc and the trough of Ginh components, whose selectivity seems inversely proportional to the frequency bandwidth in the temporal spectrum of the visual drive. **(C)** Complex cell (Cell 8) presented in Figure 12B. Spike trigger averaging (STA) and cross-correlogram (CC) for two stimuli conditions only (GEM and NI). Same conventions that in **(B)**. The patterns observed in both eye-movement animated stimuli (GEM and NI) suggest a slight shift (6 ms) in conductance peak between excitation and inhibition and a significantly longer time-course of inhibition relative to excitation. **(D)** Complex cell (cell 6) presented in Figure 3 for the two stimuli conditions (NI and DN) evoking a sparse regime. Too many spikes were observed in the two other conditions (DG and GEM) for allowing proper conductance measurement. **(E)** Simple Cell (cell 9), three conditions (DG, NI, and DN). **(F)** Simple Cell (cell 19), three conditions (DG, GEM, and NI).

To test for this interpretation, we have measured evoked conductance dynamics in 6 additional cells during one or several of the DG, GEM, and NI conditions (2 cells were recorded for all four conditions [example in Figure 12A and the other 4 for two or three out of four conditions (3 additional examples in Figure 13)]. We used here the continuous conductance measurement method in current-clamp mode, as reviewed in Monier et al. (2008). Similar qualitative conclusions were reached in current-clamp by artificially depolarizing the cell until spike inactivation, and thus increasing the visibility of inhibitory events which appear as phasic hyperpolarizing events (in Figure 12B, the upper waveform in the current clamp *Vm* trace panel can be compared with Ginh trace).

Let us first concentrate on Simple cells (cells 7, 9, and 10) illustrated in Figure 13: in the DG condition, as it could be expected, the membrane potential and the spike-triggered averages (STAs) were modulated at the driving temporal frequency (2–3 Hz) of the DG (see Cell 7 in Figure 12A). Excitation and inhibition were in anti-phase and the temporal window during which excitation was fed at each cycle without inhibition for several hundred ms. This is best seen by looking at the Gexc and Ginh conductance profiles in the bottom left panel of Figure 12A, and their cross-correlation pattern (CC) in the second left panel (from the top) in Figure 13B. A similar trend is seen in Cell 9 (Figure 13E). In contrast, in the same Simple cells, when the stimulus dynamics was enriched by broadband eye-movements animation (GEM and NI) or turned into white noise (DN), the peak of the spike-triggered average (STA) excitatory conductance and the trough of the STA inhibitory conductance tended to become almost synchronous. Figures 13E,F shows the example of Cells 9 and 10, where the stimuli with the richest temporal statistics (NI or DN) produced a highly phasic trough of inhibition, whose half-width at half amplitude ranged from 60 ms for NI to 15–20 ms for DN. Note that his inhibitory trough effect is not always seen for GEM stimuli (compare Cell 7 and Cell 10). This fast and reversible removal of inhibition occurred while riding (one cell) or not (two cells) on a slower inhibitory conductance peak, and was concomitant (for the three Simple cells) of a brief but slower peak of excitation. The top row panel in Figure 13 summarizes these findings: the systematic report of anti-correlated changes between excitation and inhibition for natural image animation supports the view that input statistics shape the selectivity of a temporally-restricted gate (“window of opportunity”) for spike initiation.

In the complex cell illustrated in Figure 12B (Cell 8), most phasic conductance increases were linked to large saccadic eye-movements, although this was not systematically the case across all cells (see RESULTS, section 5). The decomposition into Gexc and Ginh components further demonstrates, at least for this cell, that each burst of synchronous excitatory events was correlated with an inhibitory barrage which started at the same time but whose peak was delayed by a few ms. The peak values of the Gexc-Ginh cross-correlation waveforms (Figure 13C) were +0.68 and 0.66, for NI for GEM, respectively. For both stimulation regimes, inhibition fall-off was slower than that of excitation, as shown by the asymmetry of the profiles of the STA for Gexc and Ginh, where inhibition is still maintained at high level even for delays where excitation is already gone (Figure 13B). The highest SNR epochs were found correlated with the largest conductance increases (50–150%). These epochs also corresponded to the movie periods where the observed *Vm* dynamics departed from the linear prediction derived from the dense noise RF mapping, attesting for the transient recruitment of strong dynamic non-linearities.

In summary, our decomposition method revealed across cells a high diversity of patterns between excitation and inhibition. Nevertheless a common behavior was apparent for the whole population, even if for any given stimulus condition the precise pattern of Gexc and Ginh waveforms varied from cell to cell: the precise temporal interplay between excitation and inhibition depended on the statistics of the presented stimulus. Based on our sample, two patterns seemed to predominate, transient anticorrelation between changes in excitation (transient increase) and inhibition (transient decrease), or delayed (or longer tailed) inhibition peak relative to excitation peak. In Simple cells, excitation and inhibition tend to co-vary negatively and their relative peak and trough were found anti-correlated during DG, NI and DN stimulations. The interplay was more diverse across cells for GEM stimulations. Note also that, in all recorded Simple cells, these stimulus-dependent modulations arose on top of a tonic increase of Ginh (30–100%) relative to the rest conductance, which was not seen in the Complex cells illustrated in Figure 12B (cell 8) or in Figure 13D (cell 6). These features, namely the return to a high conductance state after the spike or the longer tail of the Ginh waveform, have in fact the same functional impact following the transient surge of synaptic excitation: they all contribute to the increase in spike precision and the sparsening of spiking activity, in particular for the NI condition.

Thus, whatever synaptic scenario, a common finding was observed. A rapid (in a few tens of ms range) and reversible change in the balance between excitation and inhibition created a “spiking opportunity window” whose temporal selectivity was inversely related to the dimensionality of the input. The broader the stimulus spectrum, the tighter the resulting opportunity window.

## Discussion

### Novelty of the strategy and results

The present report provides a comparative intracellular study of single V1 neuronal responses for stimuli of various complexities, including natural scene statistics. It differs from previous studies in the anesthetized cat (Haider et al., 2010; Herikstad et al., 2011) by a more complete parameterization of inputs statistics and a realistic simulation of the retinal flow produced by eye-movements.

In terms of strategy, the choice of simulating the retinal flow induced by eye-movements provides a telling comparison with the behaving and freely viewing animal (Vinje and Gallant, 2000, 2002 in monkey; Fiser et al., 2004 in ferret). The fact that we reproduce in the anaesthetized and paralyzed preparation the sparsening of cortical activity first reported in behaving animals attending to natural scenes suggests that this effect does not depend on attention and active motor exploration. This does not preclude the importance of extraretinal factors (extraocular proprioception and efferent copy) or specific neuromodulation linked with attentive behavior (Goard and Dan, 2009) in the reliability of sensory processing. However, our study demonstrates that the retinal flow dynamics themselves are sufficient to produce a sparsity effect often attributed to the attentional state and/or active sensory-motor exploration. Our working hypothesis is that the mechanisms revealed here reflect low-level computations entirely driven by the retinal flow statistics, which regulate ongoing processing without the supervision of higher cortical areas or behavior-related signaling (see also Fournier et al., 2011).

Our simulation of realistic retinal flow produced by virtual eye-movements has several advantages: (1) it ensures a maximal recording stability, which is needed for *in vivo* intracellular recordings; (2) the visual flow is controlled in a fully parameterized and reproducible way: in particular, a unique virtual eye-movement sequence was replicated, reducing across-trial variability for GEM and NI stimuli. Note that such a precise control cannot be achieved in the behaving animal, even if the retinal image is passively animated during gaze fixation along the previously recorded scanpath (Vinje and Gallant, 2000 and 2002), since fixational and tremor ocular movements are still present during fixation. Other studies in the anesthetized and paralyzed preparation have played plain commercial or movies video sequences recorded on the head of the animal, which do not allow to separate the impact of eye-movements from body and head movements (Yen et al., 2007; Herikstad et al., 2011; Kampa et al., 2011) or have flashed natural scenes which lack broadband temporal information (Tolhurst et al., 2009); (3) although only one cell is recorded at a given time, the intracellular technique gives access to the whole network-driven dynamics. The frequency-time analysis of the continuous fluctuations of the membrane potential allows us to retrieve the synchrony state of the effective functional assemblies in which the recorded cell is embedded. Thus, the combined subthreshold and spike signal analysis gives a multi-scale view of the global dynamics of the visually-driven network; (4) by replicating the same protocols for the same cell at different holding currents, we could extract the underlying conductance dynamics (Monier et al., 2008) and reveal possible scenarios of control of the spiking opportunity window by the interplay between excitation and inhibition.

The two main results are the following: (1) “Noise” is contextual: the global network state depends to a large extent on the full field viewing condition irrespective of the specificity of the local signal processed in the recorded cell RF. Our data show that membrane potential trajectories of V1 neurons in response to a full field NI stimulus are at the same time irregular and reliable, because of a lower level of stimulus-locked trial-to-trial variability (lower than for low dimension stimuli such as DG); (2) the evoked spike responses, for animated scenes, are sparse, temporally precise, and reliable.

The first result is original since no previous attempts were made to differentiate contextual Noise from the Signal. The methods used in this study offer, for the first time, comparative quantification of SNR in parametrically reproducible and controlled environments at the intracellular level. The dependence of Noise on stimulus conditions is linked partly to the full field stimulation, recruiting a modulatory effect of the “silent” receptive field surround. A previous related work by Haider et al. (2010) established that stimulation of the surround participates indeed to the decrease in trial-to-trial variability. The additional claim made here is that the Noise depends also on the global stimulus statistics, a conclusion supported by our detailed Signal Noise analysis. Although the “Noise term” is defined in the stimulus-locked condition, it refers to the variance across trials which depends on the global conductance state and the background gE/gI balance imposed by the full field stimulation.

Concerning the second result, other studies already reported that natural scene viewing in full field conditions results in increased sparsity, and recruits suppressive center-surround non-linearities (Vinje and Gallant, 2000; Haider et al., 2010). One should note however that the sparsening may be specific to excitatory cells (this study and Haider et al., 2010) and not to inhibitory interneurons where an opposite behavior has been reported (Haider et al., 2010). One study in the anesthetized cat shows that cortical responses to animated scenes, on the whole, exhibited a high degree of spike count variability, but a surprisingly low degree of spike timing variability (Herikstad et al., 2011). The observation that natural movies rarely produce net excitation levels in visual cortex comparable with those induced by gratings (Onat et al., 2011) is still consistent with sparse coding, although more indirect since based on VSD imaging. These different studies support the view that sparse encoding of natural scenes could be an hallmark of intracortical organization in higher mammals. Indeed this observation has been replicated in monkey (Vinje and Gallant, 2000) and cat (Haider et al., 2010; this study) but no strong evidence for sparseness has yet been found in rodent visual cortex (Hofer et al., 2011; Kampa et al., 2011). The case of ferret is still debatable, since, in one study, the natural scenes were flashed rather than animated (Tolhurst et al., 2009), and, in another one, no measures of reliability and sparsity were provided (Fiser et al., 2004).

### Failure of the linear model to predict subthreshold dynamics

Dense noise is classically used to probe the linear kernel of spiking receptive fields. DN seems a choice stimulus under the assumption that receptive fields can be accounted for by a cascade of separable linear and non-linear transfer functions. DN is known to be an optimal input pattern to extract the linear kernel irrespective the contextual dependence of the static non-linearity (Busgang theorem). However many studies in V1, including ours (Fournier et al., 2011), report that such a stimulus, depending on the cell, may not be always effective in triggering postsynaptic firing. In order to avoid the spurious impact of low level firing on the STA estimate of the spiking receptive field, we computed intracellular estimates of linear kernel with DN to recover the linear prediction of the response waveform without being concerned by noise contamination. Our results confirm the previous claim of accuracy of *Vm* reconstruction based on the spiking RF (Mohanty et al., 2012) when using DN stimuli only. Concerning low spatial frequencies, the DN noise stimulates equally all the spatial frequencies, so there is no a priori reason to believe that it could not estimate properly the low spatial frequencies. The response to the DG stimulus, which is exclusively composed of low spatial frequencies, was indeed well-predicted by the linear kernel, which proves that the estimation of the linear kernel in the low spatial frequency range was satisfactory. However, by using a larger variety of input statistics, our intracellular results demonstrate unambiguously that modeling the classical RF with a linear kernel invariant with the visual context is inadequate to predict full field responses to temporally rich stimuli such as natural scenes, or even gratings animated with eye-movements. Quite remarkably, and in agreement with previous studies (David et al., 2004; Touryan et al., 2005; see also Machens et al., 2004 in A1 cortex), responses to NI in our study were poorly predicted by the linear RF model (less than 11% of explained variance).

Our findings indicate that natural-like spatial and temporal statistics activate V1 non-linearities in a specific fashion. They also confirm a recent study from our lab, quantifying the dependency of both linear and non-linear kernels on input statistics (Fournier et al., 2011). In this latter paper, various simulations show that the present effects are not reproducible by simple adaptive or non-adaptive gain control models (Figure 7 in Fournier et al., 2011). Possible mechanisms underlying these non-linearities have been reported and result from the co-stimulation of the classical (CRF) and the surrounding non-classical (nCRF) parts of the receptive field (Haider et al., 2010). In this latter study, a simple data-driven computational model strongly suggests that changes in the global level of Ginh during the combined (CRF + nCRF) stimulation increases sparseness, while changes in Gexc have a predominant effect on increasing spike-train reliability.

### High reliability of evoked V1 responses

High response reliability has been reported previously at the subcortical stages (retina: Berry and Meister, 1998; LGN: Reinagel et al., 1999; Lesica et al., 2006; Butts et al., 2007; Desbordes et al., 2008, 2010). In V1, a majority of studies concluded to a high response variability co-varying positively with the response strength (Heggelund and Albus, 1978; Dean, 1981). Taken together, most studies suggest that sensory responses are more variable for cortical than for peripheral neurons. The stimulus-dependence we report intracellularly in V1 departs however from these observations. A first attempt was made to quantify the noise with a straightforward estimation of stimulus-locked variance over trials. The following trend was revealed and statistically significant: the reliability increases during NI, and conversely decreases for DG (bottom row in Figure 4; see also Figure 10B). However, this method remains very sensitive to fluctuations in the high frequency range (>100 Hz). The time-frequency SNR approach, developed further in the Results section, overcomes this problem and yields to the same conclusions. Furthermore, the decomposition in Signal and Noise components demonstrate that the highest reliability levels for SNR were attained with two types of eye-movement animated stimuli (GEM and NI), which evoke very different firing rates (high for GEM and low for NI), making a role of spiking refractoriness unlikely. In particular, during NI, high reliability coexists with sparse activity and subthreshold response reproducibility is specifically improved.

One should note, however, that the measures presented here were obtained in the anesthetized brain, a preparation choice dictated by the need for a reliable control of the visual input. A recent study (Goard and Dan, 2009) provides further evidence that reliability may be even higher during arousal. Nucleus basalis stimulation in the rat was shown to produce a decorrelation and sparsening effect on visual cortex responses to natural stimuli. Interestingly (in relation to the Vinje and Gallant study), this effect depends on cortical muscarinic acetylcholine receptor activation, and is accompanied by an increase in reliability of thalamic and cortical responses.

### Eye-movements, nonlinearities, and reliability

Our results demonstrate that realistic eye-movements dynamics improve the SNR of sensory processing in the early visual pathway independently from the proprioceptive and efferent copy signals (absent in our preparation). Nevertheless, it remains likely that extratretinal factors linked with eye-movements (Craspe and Sommer, 2008), have a functional impact on signal transmission efficacy in the intact alert behaving animal. It has long been known that eye-movements, both saccadic and fixational, are necessary for normal visual perception. Retinal stabilization leads to fading of the sensory percept (Ditchburn and Ginsborg, 1952; Coppola and Purves, 1996; Rucci and Desbordes, 2003; Martinez-Conde et al., 2006). Small amplitude fixational eye-movements, which occur more rarely in the cat than in primates (Körding et al., 2001), avoid bleaching of photoreceptors. Their suggested role is to maintain visual perception against fading (Martinez-Conde et al., 2004, 2006), and to increase visual acuity (Hennig et al., 2002; Rucci et al., 2007; Ko et al., 2010). One characteristic of fixational drift and tremor dynamics is the 1/f^2^ shape of the temporal power spectrum (Eizenman et al., 1985), which is accurately reproduced by our model.

Saccadic eye-movements during natural scene exploration in free viewing conditions induce a top-down signal preceding the acquisition of the visual target in the RF, and thus engage cortical processing toward the yet-to-be fixated object (Ito et al., 2011). This synchronization in the local field potential signal is thought to be of non-retinal origin since it is observed in visual areas, even in the dark, during voluntary eye-movements. Similar processes could apply also to micro-fixation movements (Maldonado et al., 2008; Rajkai et al., 2008; Bosman et al., 2009). Recent work (Martinez-Conde et al., 2012) indicates that real micro-saccadic eye-movements during visual fixation trigger a phasic increase in firing rate, followed by a rebound suppression which is absent when simulating virtual saccades (reproduced—as in the present study—by manipulating the retinal flow alone). Interestingly, micro-saccades without target produce a slow but still transient reduction of baseline activity. As a whole, this biphasic effect (excitation/inhibition like) reported in the alert monkey supports the hypothesis of a further improvement of a transient signal-to noise ratio during fixation, concomitant of the effective micro-saccade occurrence.

Irrespective of extraretinal modulation, we found in our study that the membrane potential reliability tended to increase after simulated saccades as compared to slower eye-movements. Several studies have shown that transient or flashed stimuli evoke non-linear, reliable, temporally precise and burst-like responses in V1 (Gawne et al., 1996; Mechler et al., 1998; Muller et al., 1999, 2001; Reich et al., 2001). Such responses may be recruited as early as the retina (Olveczky et al., 2003) or the LGN (Lesica and Stanley, 2004; Alitto et al., 2005; Denning and Reinagel, 2005). Thalamic bursts transmit information reliably (Guido and Sherman, 1998; Reinagel et al., 1999) and non-linearly (Guido et al., 1992; Lu et al., 1992; Mukherjee and Kaplan, 1995). In behaving animals, saccades and micro-saccades increase burst probability in thalamic and V1 neurons (Guido and Weyand, 1995; Martinez-Conde et al., 2000, 2002; Weyand et al., 2001). In the present study, eye-movement animated stimuli (mostly GEM) tended to evoke more burst-type events than DG and DN stimuli.

A partial explanation of our results could be that a precise feed-forward activity under NI stimulation is transferred from LGN to the layer 4 of the visual cortex through the highly synchronized excitation conveyed by thalamo-cortical projections, triggered by large saccadic eye-movements. A modeling study (Kremkow et al., submitted) demonstrates that simple thalamo-cortical mechanisms such as feedforward inhibition (see also Levy et al., 2013) and synaptic depression can account in push-pull simple receptive fields for the presence of precisely time-locked and transient post-saccadic modulation. Nevertheless, we report here strong evidences indicating the temporal structure of the thalamic flow or the filtering by the subcortical stages are not the only determinants of response precision at the cortical level. Reliable responses were not restricted to phasic contrast changes produced by large saccades. They also occurred during fixational eye-movements. Globally, spike reliability did not differ between saccadic and non-saccadic periods. Thus, the cortical network integration and its associated non-linearities are involved in the observed stimulus-dependent reliability. Our data suggest furthermore in the animated natural scene condition that some intracortical mechanism may filter out the postsaccadic modulation, which could be seen otherwise as a confusing source of correlation masking novel information from the external environment. Note that most retinal cells share the same transient activation during large saccadic exploration (“shift effect”) and that self-generated reafferent activity is transmitted to sensory structures by internal copy signals of the oculomotor activity. Similar views on perceptual filtering by efferent copy related signals have been proposed in the electrosensory system of the electric fish (Bell, 2001), which can be seen as a functional metaphor of the visuo-oculomotor system.

### Synaptic conductance interplay and spike timing precision

The present study addresses also the synaptic mechanisms underlying the temporal precision of the sensory code. Our conductance analysis reveals a diversity of temporal interplay between excitation and cortical inhibition, across cells and stimuli. However, during NI and GEM stimulations, the common finding to all cells was that the balance between excitation and inhibition changed transiently and reversibly around spike emission. This effect was present, but to a lesser extent, in DN conditions. The phasicity of the conductance changes is reminiscent of the “spiking window of opportunity,” already described in S1 (Hull and Scanziani, 2007) and A1 (Wehr and Zador, 2003; Tan et al., 2004) cortex. The attunement of the spiking opportunity window width with the broad temporal spectrum of the sensory input is likely to be the substrate of the stimulus dependence of the spiking temporal precision, as shown in Figure 6.

While delayed suppression and its role in controlling spiking precision have been studied in other cortical areas and different sensory modalities (somato-sensory: Gabernet et al., 2005; Wilent and Contreras, 2005; Okun and Lampl, 2008; auditory: Wehr and Zador, 2003), its prevalence in visual cortex remains still an open issue. Indeed, a diversity of temporal interplay has been reported ranging from in-phase, lagged, to anti-phase behavior depending on the type of stimulus and class of recorded RF (see Monier et al., 2008 for a review). Our conductance measurements during eye-movement animation (NI and GEM) show that depending on the cell the change in the excitation-inhibition balance is produced by two processes: either lagged excitation-inhibition or drop of inhibition concomitant of a surge of excitation. In both cases, this balance changes transiently during a few tens of ms, in such a way as to control the precise timing of spike initiation during a shorter efficient window of 5–10 ms. Both mechanisms force transiently V1 neurons in a coincidence detection regime. It is likely that the diversity of effects reported in terms of absolute conductance profiles reflect the hierarchical positioning of the considered postsynaptic cell in the intracortical circuitry. Different afferent circuit implementations for the same function might be found in the thalamo-recipient layers vs. the association and recurrent layers (e.g., supragranular). There could be also structural differences in phyologenetic constraints expressed in higher mammals vs. rodents. In the rat, the fine regulation of spiking reliability in sensory cortices seems to result from the “push-push” balance of excitation and (slightly delayed) inhibition, whereas in cat layer 4 visual cortex at least, “push pull” alternance between excitation and inhibition seems to be prevalent.

### Low noise coding

In agreement with theoretical predictions (Barlow, 1972; Olshausen and Field, 1996), we observed that the evoked spike patterns became sparser when stimulus statistics were changed from a drifting grating to a more complex natural movie. The pioneer extracellular work of Vinje and Gallant (Vinje and Gallant, 2000, 2002) was the first to suggest that center-surround interactions may participate to V1 activity sparsening during NI. Our intracellular experiments further show the presence of a dense and reproducible pre-synaptic bombardment (detected by the time-frequency SNR analysis) and indicate that natural statistics are well-suited to recruit suppressive non-linearities.

More generally, we propose that the non-linear spike generating mechanism, the subcortical amplification of transient stimuli, and the suppressive (including center-surround) interactions in V1 [studied in depth by Haider et al. (2010)], all constrain the number of dynamical states the *Vm* can occupy in response to a specific stimulus, provided that the input statistics belong to the spatiotemporal domain where these non-linearities are maximally active. This is the case for the NI stimulus, as indicated by the low level of Noise and the high reproducibility of evoked *Vm* trajectories even in the absence of spiking. On the contrary, the high level of Noise in the responses to GEM and DG suggests that their statistics did not recruit the full computational capacity of early visual processing. Each of these stimuli can be encoded by a multiplicity of dynamical network states, hence resulting in a high trial-to-trial variability.

## Conclusion

Our intracellular study supports previous theoretical studies proposing that V1 visual RFs are best fitted to detect natural statistics (see Simoncelli and Olshausen, 2001 for a review). Maximization of mutual information or optimization of activity sparseness have been both shown to produce realistic V1-like spatial RFs under the guidance of natural input statistics (Olshausen and Field, 1996; Bell and Sejnowski, 1997). We propose accordingly that the low noise and high temporal precision we observed in the membrane potential and spiking behavior of V1 cells reflect the following fundamental computational principle: the determinism (or reproducibility) of the full V1 network dynamics is maximized conditionally to the global statistics learned during visuomotor exploration of the natural environment.

## Summary

This *in vivo* intracellular study in cat V1 shows that (1) the noise, the temporal precision and the trial-to-trial reliability of cortical synaptic and spiking responses all depend on the visual input statistics and that (2) natural stimuli seen through simulated eye-movements evoke sparse spike responses which arise from irregular and highly reproducible membrane potential (*Vm*) trajectories.

### Conflict of interest statement

The authors declare that the research was conducted in the absence of any commercial or financial relationships that could be construed as a potential conflict of interest.
